# Endocannabinoid System and Tumour Microenvironment: New Intertwined Connections for Anticancer Approaches

**DOI:** 10.3390/cells10123396

**Published:** 2021-12-02

**Authors:** Marta Iozzo, Giovanna Sgrignani, Giuseppina Comito, Paola Chiarugi, Elisa Giannoni

**Affiliations:** Department of Experimental and Clinical Biomedical Sciences “Mario Serio”, University of Florence, 50134 Florence, Italy; marta.iozzo@student.unisi.it (M.I.); g.sgrignani@student.unisi.it (G.S.); giuseppina.comito@unifi.it (G.C.); paola.chiarugi@unifi.it (P.C.)

**Keywords:** endocannabinoid system, tumour microenvironment, immune cells, anti-cancer treatment, preclinical models

## Abstract

The tumour microenvironment (TME) is now recognised as a hallmark of cancer, since tumour:stroma crosstalk supports the key steps of tumour growth and progression. The dynamic co-evolution of the tumour and stromal compartments may alter the surrounding microenvironment, including the composition in metabolites and signalling mediators. A growing number of evidence reports the involvement of the endocannabinoid system (ECS) in cancer. ECS is composed by a complex network of ligands, receptors, and enzymes, which act in synergy and contribute to several physiological but also pathological processes. Several in vitro and in vivo evidence show that ECS deregulation in cancer cells affects proliferation, migration, invasion, apoptosis, and metastatic potential. Although it is still an evolving research, recent experimental evidence also suggests that ECS can modulate the functional behaviour of several components of the TME, above all the immune cells, endothelial cells and stromal components. However, the role of ECS in the tumour:stroma interplay remains unclear and research in this area is particularly intriguing. This review aims to shed light on the latest relevant findings of the tumour response to ECS modulation, encouraging a more in-depth analysis in this field. Novel discoveries could be promising for novel anti-tumour approaches, targeting the microenvironmental components and the supportive tumour:stroma crosstalk, thereby hindering tumour development.

## 1. The Endocannabinoid System

Thirty years after the isolation of Delta-9-tetrahydrocannabinol (Δ9-THC) and cannabinol (CBD), the two phytocannabinoids mostly represented in the inflorescences of *Cannabis Sativa*, the endocannabinoid system (ECS), a complex intercellular communication system, was identified and characterised. It is composed by bioactive lipid derivatives with binding affinity similar to THC and CBD, known as endocannabinoids (eCBs), a heterogeneous class of receptors and a complex of enzymes responsible for the synthesis, transport, and hydrolysis of eCBs.

*N*-arachidonoylethanolamine (AEA) and 2-arachidonoylglicerol (2-AG) are the first discovered eCBs while other molecules such as *O*-arachidonoylethanolamine (virodhamine), 2-arachidonyl glyceryl ether (noladin), *N*-arachidonoyldopamine, and palmitoylethanolamine (PEA) were recognised and characterised later. 

**Figure 1:**
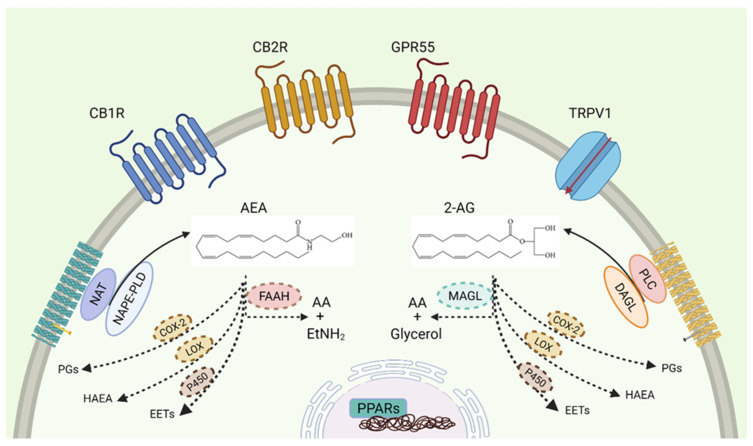
The components of endocannabinoid system. The two principal bioactive lipids (AEA, 2-AG), the receptors (CB1R, CB2R, GPR55, TRPV1, and PPARs) and the biosynthetic (NAPE-PLD or DAGL) and catabolic enzymes (FAAH, MAGL or alternative oxidising enzymes) are shown. Created with BioRender.com.

The production of eCBs begins with a synthesis “on demand” from membrane phospholipid precursors. N-acyl transferase (NAT) and N-acyl phosphatidylethanolamine phospholipase D (NAPE-PLD) are the canonical enzymes which synthesise AEA [1,2,3]. On the other hand, a first hydrolysis by phospholipase C-β (PLC-β) and a second reaction by diacylglycerol lipase (DAGL) are required for 2-AG formation [4,5,6]. Once synthetised, such lipids are metabolised by fatty acid amide hydrolase (FAAH) and monoacylglycerol lipase (MAGL), releasing arachidonic acid (AA) and ethanolamine or glycerol, for AEA and 2-AG, respectively (Figure 1). Cyclooxygenase-2 (COX-2), 5-, 12-, 15-lipoxygenase (5-, 12-, 15-LOX), and cytochrome P450 additionally direct eCBs to alternative catabolic routes, actively contributing to inflammation [7,8]. In this context, the oxygenation of AEA and 2-AG by COX-2 and LOXs can be done thanks to arachidonoyl moiety in eCBs that makes them vulnerable to these eicosanoid enzymes. So, given these considerations, it is known that the inhibition of FAAH and MAGL increases AEA and 2-AG levels, enhancing eCBs oxidation by COX-2, thus the accumulation of prostamides and prostaglandin glycerol esters (PG-Gs) [9,10].

The ECS controls many biological events, such as synaptic plasticity, neuroprotection, immune response modulation, energy homeostasis [11,12,13], and over the past twenty years, a growing number of evidence has shown that its alteration correlates with the onset of various diseases including cancer [14,15].

### 1.1. Receptors

Cannabinoid receptors (CBRs) are members of the large family of seven α-helical transmembrane G protein coupled receptors (GPCRs) activating G_i/0_ proteins [16,17]. CB1R (chromosome 6q15) is predominantly located in central nervous system where it modulates the release of neurotransmitters, playing a pivotal role in memory, motor coordination and emotional processes [18,19,20]. Some peripheral tissues, such as liver, heart, skeletal muscle, adipose tissue, and gastro-intestinal tract, also express CB1R [21,22]. On the other hand, CB2R (chromosome 1p36.11) is predominantly expressed on lymphoid organs and immune cells eliciting anti-inflammatory and immunosuppressive functions. However, it was also identified in certain regions of nervous system [23].

In addition to CB1R and CB2R, endocannabinoids also modulate other receptors and channels defined non-canonical CBRs, including GPR55 (chromosome 2q37.1), the transient receptor potential vanilloid 1 (TRPV1) (chromosome 17p13.2) and the nuclear peroxisome proliferator-activating receptors (PPARs) α and γ (chromosome 22q13.31 and 3p25.2, respectively) (Table 1).

GPR55 is a de-orphaned GPCR, known as CB3R. It shares a low sequence homology with CB1R and CB2R and, unlike them, it signals through Gα_12/13_ and Gα_q_ proteins [28,57]. Different tissues, e.g., brain, spleen, bones, adipose tissue, gastro-intestinal tract, and islets of Langerhans, were found to express GPR55 mRNA [29]. Despite the limited amount of literature about the role of this receptor, it was seen to contribute to vascular functions, bone turnover, motor coordination, and to have some implications in neuropathic/inflammatory pain, neurological disorders and metabolic/immune dysregulation [31,32,33,34,36,58,59].

Some TRP channels, beside to GCPR, belong to non-canonical CBRs. TRPV1 is a member of that class. It is a six transmembrane non-selective cation channel, particularly permeable to Na^+^ and Ca^2+^ ions [60]. It was found to be prominently expressed in neurons of dorsal root, trigeminal, vagal ganglia, in arteriolar smooth muscle cells, and in bladder urothelium where physical/chemical stimuli, including temperatures over 43 °C, acidic conditions (pH < 6), and vanilloids can prompt its activity [43,44,45,46,47]. TRPV1 detains a principal role in thermoregulation, pain but it was also found to be implicated in cough and bladder hyperactivity [47,61,62,63].

Several evidence reported the involvement of cannabinoid receptors in cancer, highlighting the different pro- and anti-tumour actions that can be mediated by CBRs depending on the ligand, cancer tissue and other environmental factors.

Alterations of CB1R and CB2R expression levels and/or function have been observed in several cancer types. High levels of CB1R were found, for example, in invasive ovarian tumours, in cancers of the digestive tract and in Hodgkin lymphoma cells [24,64,65]. In breast tumours elevated CB2R levels correlated with malignancy [66]. High CB2R expression correlated with the presence of metastases in the lymph node and with the greatest risk of cancer recurrence in malignant thyroid lesions. In prostate and lung carcinomas, CB1R and CB2R are often upregulated, particularly in prostate where their levels increase with the higher degree of malignancy [25,26,27].

**Figure 2:**
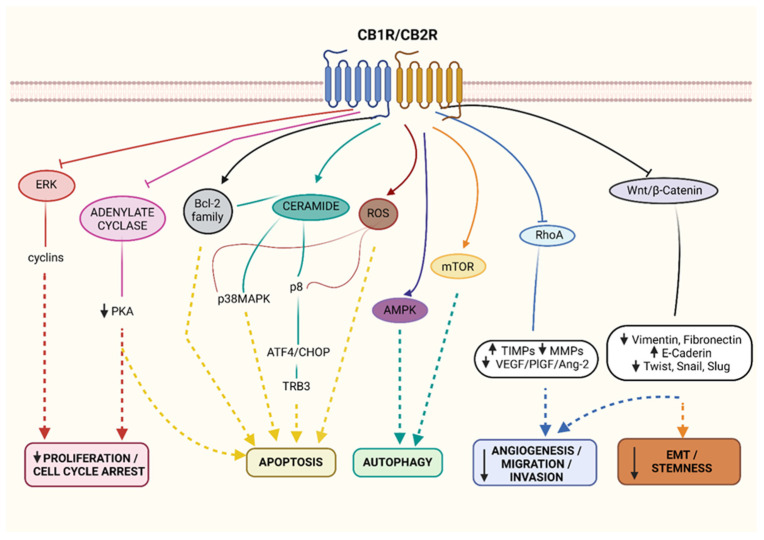
Canonical CBRs signalling in cancer. Cannabinoids inhibit proliferation and cell cycle (↓ERK/cyclins; ↓Adenylate cyclase/PKA), induce apoptosis (↑Bcl-2; ↑p38MAPK; ↑p8/ATF4/CHOP/TRB3; ↑ROS) and autophagy (↑AMPK; ↑mTOR), decrease angiogenesis (↓VEGF/PIGF/Ang-2), migration and invasion (↓RhoA; ↓MMPs). The modulation of CB1R/2 also reduce EMT (↓Vimentin, Fibronectin) and stemness (↓Wnt/β-catenin; ↓Twist, Snail and Slug). Created with BioRender.com.

Considering all the potential implications arising from the modulation of CB1R and CB2R signalling pathways, the targeting of these receptors is becoming a new anti-cancer strategy. Indeed, CB1R and CB2R drives anti-proliferative and pro-apoptotic effects through inhibition of adenylate cyclase and the consequent decrease in cyclic Adenosine Monophosphate (cAMP)/cAMP-dependent protein kinase (PKA) activity [67]. Moreover, the block of the extracellular signal regulated kinase (ERK) activates CB1R/CB2R-cell proliferation arrest. CB1R and CB2R upregulate programmed cell death activating Bcl-2 family, increasing reactive oxygen species (ROS) generation and de novo synthesis of ceramide (as reviewed in [27]). Specifically, the pro-apoptotic sphingolipid ceramide is a trigger for p38 mitogen-activated protein kinase (MAPK) pathway and for the upregulation of the endoplasmic reticulum (ER) stress regulated protein 8 (p8), therefore resulting in an increased expression of the activating transcription factor 4 (ATF4), C/EBP homologous protein (CHOP), and the stress-related pseudo-kinase tribbles homolog 3 (TRB3) [68]. CB1R and CB2R also activate autophagy, downregulate cell migration, angiogenesis, and impair epithelial-to-mesenchymal transition (EMT) of cancer cells. Autophagy occurs via mammalian target of rapamycin (mTOR) and AMP-activated protein kinase (AMPK) [69]. On the other hand, reduction in cell migration and angiogenesis is elicited through the inhibition of the RhoA-focal adhesion kinase (FAK)-proto-oncogene tyrosine-protein kinase (Src) axis which induces the release of tissue inhibitor matrix metalloproteinases-1 (TIMP-1) and downregulates proangiogenic factors, such as vascular endothelial growth factor (VEGF), placental growth factor (PlGF), and angiopoietin-2 (Ang-2). Additionally, CBRs prevent EMT by interfering with the Wnt/β-catenin pathway (Figure 2) (review in [27]).

For what concerns GPR55, it was found in several cancer types, such as glioma, melanoma, breast, prostate, ovarian and pancreatic cancer [38,39,40]. In glioma and pancreatic cancer, pharmacological and genetic inhibition of the receptor was found to reduce tumour cell growth [38,70]. In breast cancer, the activation of GPR55 stimulated pro-invasive features by influencing migration of human breast cancer cells [37,71,72]. Moreover, in melanoma, the receptor influenced the viability of A375 cell lines [73]. It signals through Gα_q_ subunit which stimulates PLC activity, resulting in the release of diacylglycerol and Ca^2+^ from the ER and thus in the activation of different isoforms of protein-kinase c (PKC) [32,74]. These, in turn, catalyse the phosphorylation of various targets, such as the MAPK/ERK proteins, among which ERK1/2 that impacts on gene expression through the activation of the transcription factors CREB (cAMP response element-binding protein) and NF-kB (nuclear factor kappa-light-chain-enhancer of activated B cells) [30]. On the other hand, signalling through the Gα_12/13_ pathway, GPR55 can lead the RhoA/ROCK pathway, further regulating PLC activity, actin cytoskeleton and p38/ATF2 [75]. As is well-known, MAPKs and RhoA/ROCK pathways are able to orchestrate a plethora of cellular functions including proliferation, division, differentiation, apoptosis and cytoskeleton remodelling. Moreover, both signalling were found deregulated in various cancers [29].

**Figure 3:**
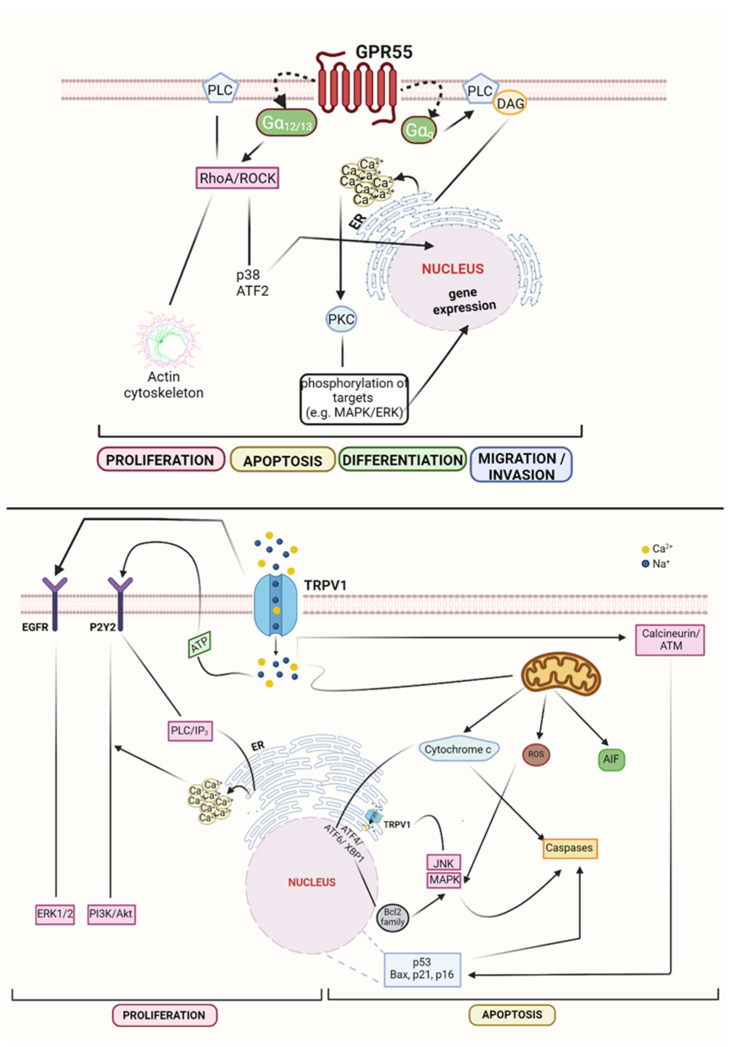
Non-canonical CBRs signalling in cancer. GPR55 modulation leads to proliferation, apoptosis, differentiation, and migration, through different molecular pathway. TRPV1-dependent mechanisms induce proliferation (↑ERK1/2; ↑PI3K/Akt), or apoptosis (↑cytochrome c/caspases; ↑ROS/JNK/MAPK; ↑AIF; ↑p53/Bax/p21/p16). Created with BioRender.com.

TRPV1 is also expressed in several neoplasms, among which primary brain tumours, pancreatic, breast, prostate and squamous cell carcinomas of the human tongue, although its role in the tumour evolution is still not so clear [49,50,51,52,76]. However, it is known that activation of TRPV1 can influence the balance between cell proliferation and apoptosis, depending on Ca^2+^ and Na^+^ influx into the cytosol [77]. In detail, proliferation can be resulted from Ca^2+^ entry, ATP release and from the transactivation of epidermal growth factor receptor (EGFR). ATP can bind to the membrane P2Y2 receptor, triggering the PI3K/Akt pathway and upregulating, via PLC, the inositol 1,4,5-trisphosphate (IP_3_), which causes Ca^2+^ release from the ER. Moreover, the transactivation of EGFR, prompts Ras/Raf/MAPK-ERK kinase (MEK)/ERK1-2 pathway, promoting cell proliferation together with Akt. Conversely, mechanisms that activate apoptosis occur through mitochondria membrane depolarisation, ER stress, nucleus and cytosol. The first event is driven by Ca^2+^ and Na^+^ influx into the mitochondria which then releases cytochrome c. On the other hand, ER stress led to c-Jun N-terminal kinase (JNK) release into the cytosol and to the upregulation of the nuclear transcription factors ATF4, ATF6, and X-box binding protein (XBP), which decrease Bcl-2. In addition, Ca^2+^ entry through TRPV1 in cytosol, activates calcineurin and ataxia-telangiectasia mutated kinase (ATM) to finally upregulate p53 and consequently Bax, p16^INK4A^ and p21. These last three factors together with cytochrome c, activates caspase 9 and 3, causing apoptosis (Figure 3) [78,79,80,81,82,83,84,85].

PPARα and PPARγ represent novel discovered CBRs. PPARα is widely distributed in metabolically active tissues (e.g., liver, heart, and muscle), controlling fatty acid catabolism and inflammatory processes, while PPARγ (isoform -γ1, -γ2, and -γ3) is differentially expressed in tissues (-γ1 ubiquitous; -γ2 in adipose tissue; -γ3 in macrophages) and involved in adipocyte formation, insulin sensitivity, inflammation [53,86]. Fibrates and thiazolidinediones are the main ligands of PPARα and PPARγ, respectively, and they are used clinically in the treatment of hyperlipidemia (fibrates) and in the treatment of type 2 diabetes (thiazolidinediones). To date, it is known that several cannabinoids can activate these receptors (e.g., PEA, OEA activate PPARα; e.g., THC, AEA and 2-AG activate PPARγ) and that PPARs have an involvement in cancer (e.g., colon, ovarian, breast and prostate cancer) [55,56]. However, many aspects have to be clarified and, therefore, there is a clear need for additional studies in this context.

Interestingly, recent discoveries pointed out that CBRs can create heterodimers with other receptors. In particular, it is known that GPR55 dimerises with CB2R in several tumours such as breast cancer, with implications in cancer malignancy [87]. Moreover, these heteromers are over-expressed in bones and hematopoietic cells, probably regulating cancer-related processes. CB2R can also be coupled to CXCR4 in breast and prostate cancers where the heteromers impact on cell proliferation, adhesion, invasion, and metastatic processes. Therefore, it was found that CB2R agonists can reduce CXCR4 activity and hinder the effects resulting from CXCR4-agonists [88,89]. A recent known hallmark of cancer is the heteromers CB2R-HER2, typical of breast cancers. In this context, it was demonstrated that cannabinoid agonists at CB2R can lead to disruption of these heteromers, hampering HER2 activity (discussed below) [66,90].

### 1.2. Cannabinoid Receptor Agonists

Cannabinoid receptor agonists represent a group of phyto-, endo-, and synthetic cannabinoids able to bind cannabinoid receptors with different affinities and efficacies. Based on their chemical structure, they are distinguished into classical, non-classical compounds, aminoalkylindoles, and eicosanoids [91].

Δ9-THC, Δ8-THC (naturals), and 11-hydroxy-Δ8-THC-dimethylheptyl (HU-210) (synthetic) are the principal classical molecules and consist of tricyclic dibenzopiran derivatives. They bind both CB1R and CB2R, specifically HU-210 with greater affinity than the natural cannabinoids.

Non-classical ligands share the similar bicyclic and tricyclic structure of Δ9-THC, lacking the pyran ring. Among these, CP 55,940 works as a full agonist at both CBRs, acting in a nanomolar range of affinity. Although this compounds directly target both CB1R and CB2R, the higher specificity for CB2R represent a molecular approach to overcome the psychotropic effects given by CB1R. Indeed, CB2R selective agonists, such as JWH-133 (classical), JWH-015, and AM1241 (aminoalkylindoles), are becoming optimal candidates for anticancer therapy.

The cannabimimetic aminoalkylindole WIN 55,212-2 shares an intrinsic activity for both CBRs similar to CP 55,940 and HU-210, however it showed to be more specific for CB2R [92,93].

For what concerns eicosanoids, the class principally includes AEA and 2-AG, endogenous ligands for CB1R and CB2R, although AEA is a partial agonist for CB2R with respect to 2-AG [91]. Together with L-α-lysophosphatidylinositol (LPI) and virodhamine, AEA and 2-AG modulate the activity of GPR55 [94]. Moreover, AEA is considered a full agonist (endovanilloid) at TRPV1 in different tumour cell lines, while 2-AG is weakly active on TRPV1 [95]. Finally, it has been also reported that AEA and 2-AG activate PPARα and -γ [96].

Although still under study, the anti-cancer activity of several CBRs’ agonists has now been assessed in many types of tumours (Table 2). Most of them exert anti-proliferative and apoptotic outcomes by activating different receptors and signalling pathways dependent on the specific cancer types (see the table for detail). A reduction in the migratory/invasive potential of cancer cells was also reported for some CBR agonists, mainly driven by the inhibition of metalloproteinases.

### 1.3. Other Agonists

R(+)-Methanandamide and Metfluoroanandamide (Met-F-AEA) are two non-hydrolysable and metabolically stable AEA analogues, with main affinity for CB1R. In prostate and human cervical cancer cells, R(+)-Methanandamide exerted anti-proliferative and pro-apoptotic functions, respectively [97,98]. The compound induced an arrest of cell cycle in G0/G1 phase and induced necrosis in gastro-intestinal cancer in vitro [99]. In breast cancer Met-F-AEA inhibited cell cycle, tumour cell adhesion and migration, interfering with the RhoA/ROCK signalling pathway and FAK phosphorylation [100,101,102]. In lung, gastro-intestinal and thyroid tumours apoptosis, and cell growth were modulated [103,104,105]. The inhibition of cell growth was also reported in melanoma in vitro [106].

PEA is an endogenous fatty acid amide related to AEA. It principally agonises PPARα and also activates GPR55 and TRPV1, with modalities yet to be clarified. Moreover, it is known that PEA is an indirect activator of CBRs, as inhibitor of FAAH which increases AEA and 2-AG levels [107]. It induced cell death in high-grade astrocytoma/neuroblastoma cells, it slowed down melanoma cell survival; moreover, it potentiated the cytotoxic effect of AEA on human breast cancer in vitro [52,108,109].

ACEA (Arachidonyl-2-chloroethylamide) is a CB1R-selective agonist. It activated apoptosis in colon cancer cells through Tumour Necrosis Factor-α (TNF-α)-mediated ceramide de novo synthesis [110]. In hepatocellular cancer (HCC) ACEA downregulated cell viability, invasion as well as MMP-2 and MMP-9 expression [111]. Moreover, invasion was also blocked in breast cancer stem cells [112]. In pancreatic cancer ACEA induced ROS-mediated autophagy via activation of AMPK, inhibition of energetic metabolism; it decreased Glyceraldehyde 3-phosphate dehydrogenase (GAPDH) and Pyruvate Kinase M2 (PMK2) expression and enhanced the anticancer potential of gemcitabine [113,114].

As aforementioned, JWH-015 and JWH-133 are selective for CB2R. In PC-3 prostate cancer, metastatic breast cancer MCF-7 cells and lung cancer cell lines, JWH-015 had anti-proliferative action; moreover, it inhibited the activation of EGFR in ERα breast cancer cells [98,115,116]. In lung cancer the compound attenuated growth factor-direct in vitro chemotaxis and chemo-invasion by reducing focal adhesion complexes, inhibiting Akt phosphorylation and MMP-9 expression/activity [25]. JWH-133 counteracted proliferation and migration of glioma, breast cancer cells and decreased trans-endothelial migration of melanoma cells [117,118,119].

### 1.4. Cannabinoid Receptor Antagonists/Inverse Agonists

Due to the “double face” of cannabinoid receptors, that are not only involved in tumour suppression but also in tumour development and progression, research in the field of cannabinoids has progressed further towards the synthesis of receptor antagonist compounds (Table 3). In this regard, SR141716 (Rimonabant) and CBD are well-known CB1R antagonists. Specifically, CBD can also act as inverse agonist/negative allosteric modulator at CB1R and partial agonist at CB2R [120,121]. In addition, several reports demonstrated that it can activate TRPV1, PPAR-γ and antagonise GPR55 [122,123,124]. AM251 and 6-iodopravadoline (AM-630) are two synthetic cannabinoid CB2R inverse agonists. However, AM251 is also reported to activate GPR55 [125]. CID16020046 is a selective GPR55 antagonist recently used in the anti-cancer research.

In breast cancers SR141716 blocked cell proliferation, effect also found in gastro-intestinal tumours where the compound, after arresting tumour cell population in G2/M phase, induced mitotic catastrophe [126,127]. In glioma cells SR141716 activated caspase-dependent apoptosis via G1 phase stasis and it up-regulated the cell membrane MICA/B, a potent a stress-induced ligand for the natural-killer group 2, member D (NKG2D) receptor, expressed in NKs [128]. Moreover, Fiore et al. reported that SR141716 impacted in chemoresistance and cancer stemness through inhibition of Wnt/β-catenin pathway in primary colon cancer stem cells (CSCs) [129].

CBD induced apoptosis pathways in gastro-intestinal cancers through excessive ROS production, ER stress and Noxa activation [130,131]. Apoptosis linked to ER-stress was also reported in breast, prostate and in GBM [132,133,134]. Interestingly, in lung and breast cancers CBD stimulated apoptosis by direct and indirect regulation of PPAR-γ [135,136]. In breast cancer, CDB induced a crosstalk between apoptosis and autophagy modulating cancer cell death [137]. CDB induced cell cycle arrest and reduction in cell proliferation in gastro-intestinal, breast, prostate and in brain cancers [130,133,138]. The reduction in cell migration and invasion was reported in gastro-intestinal cancer, lung cancer, and breast cancer [138,139,140,141,142]. In GMB and glioma, CBD reduced cell invasion through inhibition of Id-1 expression and downregulation of proteins specifically involved in growth, invasion and angiogenesis (e.g., MMP-9, TIMP-4, VEGF, TGF-β) [143,144]. Moreover, CBD increased the uptake of doxorubicin (DOXO) in breast cancer cells, it detained synergistic anti-proliferative effects with docetaxel and/or bicalutamide in prostate cancer cells, it increased chemosensitivity to Temozolomide (TMZ), Carmustine (BCNU), and DOXO in glioma [134,145,146]. However, in glioma, CBD failed to exert good cytotoxicity if compared to its activity in combination with HSP inhibitors [147].

AM251 decreased proliferation and migration in renal cell carcinoma. Moreover, it induced apoptosis by upregulating Bax and decreasing Bcl-2 expression [148]. Induction of apoptosis was also addressed in pancreatic cancer via receptor independent mechanisms. Despite these effects, it was fond that the compound reverts the anti-tumour activities of Met-F-AEA and ACEA, in gastro-intestinal and breast cancer, respectively, impacting on proliferation and cancer cell potential [104,112]. Few evidences are reported for AM-630 and CID16020046 in tumours, however it is known that AM-630 induced cell cycle arrest in G2/M phase and it inhibited migration in renal cell carcinoma [149]. In gastro-intestinal cancer CID16020046 inhibited cancer cell growth through downregulation of ERK1/2 phosphorylation and it decreased migration and the ability of adhesion to endothelial cells [150,151]. In breast cancer the compound reduced migration and chemoresistance through downregulation of multidrug resistance exporters, such as the breast cancer resistance protein (BCRP) [152].

### 1.5. Cannabinoid Enzymatic Dysregulations in Cancer

Synthesis and degradation of endocannabinoids were found deregulated in several malignant tissues. Downregulation and altered activity of NAPE-PLD and FAAH, followed by a reduction in AEA, was found in glioma tissues where, on the contrary, increased 2-AG levels correlated with a decrease in MAGL and an unchanged DAGL-α expression [153]. In endometrial carcinoma, AEA amounts were higher with respect to healthy tissues, as a consequence of FAAH reduction and NAPE-PLD increase [154]. On the contrary, in prostate adenocarcinomas biopsies FAAH protein expression increased in comparison to non-tumour biopsies [155]. In HCC MAGL expression was higher in patient with poor prognosis [156]. To date, MAGL and FAAH inhibitors are the most studied cannabinoid enzymatic targeting strategies.

### 1.6. MAGL Inhibitors

JZL184 and URB-602 are two examples of MAGL inhibitors. Nomura et al. attested that JZL184 impacted on PC3 cell migration, invasion, and survival [157]. In CRC cell lines, the compound decreased proliferation, increased apoptosis (by regulating the expression of Bcl-2 and Bax), and it improved tumour cell sensitivity to 5-fluorouracil. Moreover, in these tumour cells, it suppressed migration and altered the expression of EMT markers for example increasing E-cadherin, decreasing Vimentin and the Snail family transcriptional repressor 1 (SNAI1) [158]. Zhang et al. demonstrated that JZL-184 reduced HCC proliferation, apoptosis and invasion in vitro, suggesting that MAGL activated both proliferation and invasion of HCC cells through unknown mechanisms involving the prostaglandin E2 (PGE2) and lysophosphatidic acid (LPA) [159]. In osteotropic breast and prostate cancer cells, JZL184 reduced migration and invasion. Moreover, the compound blocked the ability of these cells to stimulate osteoclastogenesis in co-cultured models. JZL184 also inhibited the differentiation of primary osteoblast and early differentiated osteosarcoma cells. In vivo it prevented the formation of bone nodule in the presence and absence of the highly metastatic osteosarcoma cells. Given the evidence, JZL184 could therefore be considered an optimal candidate for the treatment of cancer-associated bone diseases. However, it exerted paradoxical reduction in bone volume via an effect that involved the activation of the skeletal endocannabinoid system. Further investigations are therefore needed in this context [160]. For what concerns URB-602, it inhibited tumour growth and angiogenesis in xenograft models of colon carcinogenesis. Moreover, it attenuated azoxymethane-induced pre-neoplastic lesions, polyps and tumours in vivo [161]. Accordingly, González et al. reported that the compound, given with Cannabigerol (CBG), weakly antagonist at CB1R, and O-1602 (synthetic GPR55 agonist) induced apoptosis, reduced angiogenesis, tumour volume, and aberrant crypt foci (ACF) on colorectal cancer (CRC) models in vivo [162].

### 1.7. FAAH Inhibitors

In lung cancer, the two FAAH inhibitors Arachidonoyl serotonin (AA-5HT) and URB597 reduced invasion of A549 cells, upregulating TIMP-1. Moreover, the compounds inhibited metastatic processes in vivo [163]. Always in lung cancer, Ravi et al. reported that URB597 downregulated cyclin D1 and CDK4, it activated apoptosis (via caspase-9 and PARP) and it inhibited MMP-2 and stress fibre formation [103]. In non-small lung cancer (NSCLC) URB597 in combination with Met-F-AEA significantly reduced in vitro EGF-induced proliferative and chemotactic activities with respect to Met-F-AEA given alone. Moreover, in combination with PEA, the compound decreased B16 melanoma cancer cell viability [108].

**Table 2:** In vitro evidence of the main cannabinoid receptor agonists in different tumour subtypes.

COMPOUND	TUMOUR	ACTION	REF.
**∆9-THC** **(classical)**	Gastro-intestinal cancer	Induction of apoptosis through CB1R-mediated inhibition of RAS-MAPK/ERK and PI3K-Akt survival signalling cascades	[164]
	HCC	Anti-proliferative action associated with accumulation of ceramide, ER-stress and PPARγ activity Autophagy-mediated cell death in combination with JWH-015	[69,165]
	Lung cancer	Inhibition of tumour cell growth (reduction in 3H thymidine and 14C-uridine uptake) Inhibition of EGF-induced proliferation/migration and invasion, reduction in EGF-induced phosphorylation of ERK1/2, ERK1/2 and Akt	[166,167]
	Breast cancer	Disruption of HER2-CB2R heteromers leading to HER2-proteasome degradation Induction of cell cycle arrest through Cdc2 downregulation, leading to apoptosis Reduction in 17β-oestradiol-induced proliferation	[90,168,169]
	Prostate cancer	Induction of apoptosis independent from CBRs	[170]
	Pancreatic tumour	Induction of apoptosis through de novo synthesis of ceramide and consequent upregulation of ER stress related genes p8, ATF-4 and TRB3	[171]
	Brain cancer	Inhibition of cell proliferation, induction of cycle arrest, ROS production and apoptosis, given alone or in combination with CBD Autophagy-mediated cancer cell death Inhibition of MMP-2 expression and cell invasion in cultured glioma cells via ceramide accumulation and activation of p8 stress protein Increase in radiosensivity in combination with CBD	[132,172,173,174,175]
	Endometrial cancer	Inhibition of migration through down regulation of MMP-9	[176]
	Leukaemia	Induction of apoptosis via MAPK pathway Reversion of multidrug resistance together with CBD Sensitisation to cytotoxic effects of chemotherapy	[177,178,179]
	Melanoma	Induction of cell cycle arrest through Akt inhibition, activation of autophagy-mediated apoptosis	[180,181]
**WIN 55,212-2** **(aminoalkyndole)**	Gastro-intestinal cancer	Inhibition of cell proliferation and induction of apoptosis. Inhibition of Akt, downregulation of MMP-2 and VEGF-A Inhibition of cell migration/invasion and EMT markers through COX2 downregulation	[182,183,184]
	Prostate cancer	Inhibition of cell growth, induction of apoptosis, decrease in AR, PSA, PCNA and VEGF in LNCaP Prevention of neuroendocrine differentiation of LNCaP by inhibition of PI3K/Akt/mTOR axis and stimulation of AMPK	[185,186]
	Renal carcinoma	Inhibition of proliferation and cell viability. Induction of G0/G1 cell cycle arrest, apoptosis and reduced proliferation into 3D spheres	[187]
	Osteosarcoma	Inhibition on cell migration with reduction in MMP-2 and MMP-9	[188]
	Lung and testicular cancer	Induction of apoptosis	[189]
**AEA** **(eicosanoid)**	Gastro-intestinal cancer	Induction of G0/G1 cell cycle arrest and apoptosis Reduction in cell proliferation through activation of Wnt5a non-canonical pathway Inhibition of cell proliferation induced by FAS-death receptor translocation in lipid rafts, mediated by GPR55 activation	[99,190,191]
	Lung cancer	Reduction in tumour cell spreading, mimicking the anti-invasive action of FAAH inhibitors (same effect given by 2-AG, OEA, PEA)	[163]
	Breast cancer	Inhibition of cell proliferation through downregulation of adenylate cyclase and activation of MAPK, exerting downregulation on prolactin and tyrosine kinase levels	[192,193,194]
	Prostate cancer	Reduction in EGF-induced cell proliferation, induction of apoptosis and necrosis through EGFR downregulation Induction of apoptosis mediated by activation of ERK and inhibition of AKT signalling pathways (same effect given by 2-AG and Met-F-AEA)	[195,196]
	Non-melanoma skin cancer	Induction of apoptosis mediated by oxidative stress and CBR-independent signalling	[197]
	Lymphoma	Reduction of tumour cell viability	[198]
**R(+)-Methanandamide** **(stable AEA analogue)**	Prostate cancer	Inhibition of cell growth in prostate cells (PC-3)	[98]
	Cervical cancer	Activation of apoptosis mediated by COX-2 and subsequent prostaglandins synthesis via PPARγ	[97]
	Gastro-intestinal cancer	Induction of G0/G1 cell cycle arrest and necrosis	[99]
**Met-F-AEA** **(stable AEA analogue)**	Breast cancer	Induction of cell cycle arrest correlated with Chk1 activation, Cdc25A degradation and downregulation of Cdk2 activity Inhibition of adhesion and migration, interfering with the RhoA/ROCK signalling pathway and FAK phosphorylation	[100,101,102]
	Melanoma	Inhibition of cell growth	[106]
	Lung cancer	Induction of G0/G1 cell cycle arrest leading to apoptosis (in combination with UR597)	[103]
	Gastro-intestinal cancer	Increase in AEA availability, induction of oestrogen receptor β expression, decrease in proliferation rate due to CB1 up-regulation through the transcriptional activation of CNR1 promoter (CRC)	[104]
	Thyroid cancer	Induction of apoptosis via p53 and p21	[105]
**PEA**	Brain cancer	Induction of cell death	[52]
	Melanoma	Reduction of melanoma cell survival in combination with URB597	[108]
	Breast cancer	Increase in cytotoxic effect of AEA	[109]
**ACEA**	Gastro-intestinal cancer	Activation of apoptosis through TNF-α–mediated ceramide de novo synthesis	[110]
	HCC	Reduction of cell viability, invasion and MMP-2/MMP-9 expression	[111]
	Breast cancer	Inhibition of invasion in breast cancer stem cells	[112]
	Pancreatic cancer	Induction of ROS-mediated autophagy via activation of AMPK, inhibition of energetic metabolism. Decrease in GAPDH and PMK2 expression. Increase the anticancer potential of gemcitabine	[113]
**JWH-015**	Prostate cancer	Inhibition of cell growth and apoptosis induction via de novo synthesis of ceramide. Signalling pathways include JNK activation and Akt inhibition.	[98]
	Breast cancer	Reduction of tumour growth, chemotaxis and wound healing. (block of the chemokine receptor CXCR4 signalling) Inhibition of EGFR activation in ERα breast cancer cells	[115,199]
	Lung cancer	Attenuation of growth factor-directed in vitro chemotaxis and chemo-invasion. Reduction in focal adhesion complex. Inhibition of Akt phosphorylation and reduction in MMP-9 expression and activity	[25]
**JWH-133**	Brain cancer	Inhibition of glioma cell viability	[118]
	Breast cancer	Decrease in cell proliferation, induction of apoptosis, inhibition of cell migration	[119]
	Melanoma	Decrease in trans-endothelial migration in vitro	[117]

**Table 3:** In vitro evidence of the main cannabinoid receptor antagonist/inverse agonists in different tumour subtypes.

COMPOUND	TUMOUR	ACTION	REF.
**SR141716** **(CB1R selective antagonist)**	Gastro-intestinal cancer	Induction of G2/M cell cycle arrest and mitotic catastrophe Synergic effect in combination with oxaliplatin, blocking cell proliferation Impact in chemoresistance and cancer stemness, retain of architecture and heterogeneity of human healthy organoids in ex vivo cultures through inhibition of Wnt/β-catenin canonical pathway	[126,129,200,201]
	Brain cancer	Induction of cell proliferation arrest, caspase-dependent apoptosis and upregulation of the NKG2D ligand MICA/B	[128]
	Breast cancer	Inhibition of cell proliferation via CB1R-interaction with lipid rafts	[127]
**CBD** **(antagonist, inverse agonist and negative allosteric modulator at CB1R/partial agonist at CB2R)**	Gastro-intestinal cancer	Induction of G0/G1 cell cycle arrest through downregulation of CDK2-cyclin E. Activation of mitochondrial-dependent apoptosis pathway by increasing ROS production Reduction of cell migration Protection of DNA from oxidative damage, increase in endocannabinoid levels, reduction in proliferation through CB1R, TRPV1 and PPARγ involvement. Reduction of invasion and cell migration Induction of apoptosis through excessive ROS production, ER stress and Noxa activation	[130,131,139,151]
	Lung cancer	Induction of PPARγ dependent apoptosis through increased levels of COX2-dependent prostaglandins Reduction in cell migration accompanied with decreased PAI-1 Induction of ICAM-1 in cancer cells leading to lymphokine-activated killer (LAK) cell-mediated cytotoxicity Upregulation of ICAM-1 and TIMP-1 levels, decreasing cell migration via CBRs, TRPV1 and p42/44 MAPK	[135,140,141,202]
	Breast cancer	Induction of a crosstalk between apoptosis and autophagy in mediating cancer cell death Inhibition of cell proliferation, induction of apoptosis, ER stress (MDA-MB-231). Induction of cell cycle arrest at G1/S phase (MCF-7) via CBRs or TRPV1 receptors Induction of apoptosis through downregulation of mTOR, cyclin D1 and upregulation of PPARγ (T47-D, MDA-MB-231) Inhibition of EGF-induced cell proliferation, colony formation, migration and invasion. Downregulation in cytokine production Reduction of proliferation and invasion through Id-1 downregulation Increase uptake of DOXO and induction of apoptosis, via activation of TRPV2 (TNBC)	[133,136,137,138,142,145]
	Prostate cancer	Cytotoxic effects and downregulation of CB1R, CB2R, VEGF, PSA, IL-6, IL-8 in LNCaP. Reduction of spheroid formation in LNCaP stem cells Cytotoxic activity, cell cycle arrest, apoptosis induction. Induction of apoptosis in LNCaP partially due to TRPM8 antagonism and accompanied by downregulation of AR, p53, elevated ROS. Synergistic anti-proliferative effects with docetaxel and/or bicalutamide in DU-145 and/or LNCaP cells	[134,203]
	Brain cancer	Inhibition of cell proliferation, modulation of cell cycle, increase in ROS levels and apoptosis when given in combination with ∆9-THC Increase in ROS production derived from upregulation of HSP super family genes. Decrease in cytotoxic effects through HSP upregulation. HSP inhibitors in combination with CBD lead to increased cytotoxicity respect to CBD alone Inhibition of cell invasion through Id-1 downregulation Inhibition of cell proliferation and invasiveness through downregulation in proteins specifically involved in growth, invasion and angiogenesis, downregulation of ERK, Akt, and HIF-1α Inhibition of cell proliferation, induction of apoptosis and chemosensitivity to TMZ, BCNU, and DOXO through TRPV2 activation	[132,143,144,146,147]
**AM251** **(CB1R inverse agonist/GPR55 agonist)**	Pancreatic cancer	Induction of apoptosis via receptor-independent mechanisms	[204]
	Gastro-intestinal cancer	Reversion of the Met-F-AEA anti-proliferative effect	[104]
	Breast cancer	Reversion of the effect of ACEA on the decrease in the invasive potential of breast cancer stem cells	[112]
	Renal cell carcinoma	Decrease in proliferation, induction of apoptosis by upregulating Bax and decreasing Bcl-2. Inhibition of cell migration	[148]
**6-iodopravadoline (AM-630)** **(CB2R inverse agonist)**	Renal cell carcinoma	Inhibition of cell proliferation, induction of cell cycle arrest in G2/M phase, anti-migratory effects	[149]
**CID16020046** **(selective GPR55 antagonist)**	Gastro-intestinal cancer	Decrease in migration and adhesion to endothelial cell	[151]
		Inhibition of cell proliferation and ERK1/2 phosphorylation	[150]
	Breast cancer	Decrease filopodia formation and migration	[72]
		Reduction in chemoresistance through downregulation of MDR (e.g., BCRP)	[152]

## 2. The Tumour Microenvironment

The TME represents the entire ecosystem adjacent to the cancer cells, consisting of the extracellular matrix (ECM), blood vessels and supporting cells, including immune cells (T and B lymphocytes, natural killer (NK) cells, dendritic cells (DCs), tumour-associated macrophages, etc.), cancer-associated fibroblasts (CAFs), endothelial cells (ECs), adipocytes, and pericytes [205,206,207,208,209].

The TME participates through dynamic interactions to neoplastic development, actively contributing to a plethora of phenomena, such as proliferation, angiogenesis, invasion, immune escape, and metastatic spread [210,211,212].

ECM is one of the major components of the TME. The composition of the ECM can undergo substantial variation among different tumours/tissues, as well as during tumour evolution, being susceptible to variation in protein abundance, glycoproteins, and proteoglycans composition and/or ECM stiffness [213]. In addition to its supporting role, ECM is also directly and indirectly involved in different aspects of cancer cell progression, i.e., cell survival, proliferation, adhesion, and migration [214]. Moreover, ECM contributes to the development of blood vessels to supply the tumour mass and to support the metabolic processes [215]. The establishment of an hypoxic environment also concur to promote the HIF-dependent VEGF expression, resulting in an abnormal development of neo-angiogenesis and in the suppression of the anti-tumour immune response [216,217]. ECs also release angiocrine factors and promote a rearrangement of vessel architecture characterised by altered permeability which is crucial for tumour cells metastatic spreading [218,219].

Among the cellular components of the TME, CAFs exert a prominent role in several tumours. CAFs establish a biunivocal crosstalk with cancer cells and other accessory cells, mainly acting through the release of an altered secretome and producing a large amount of growth factors, cytokines, chemokines, and metabolites, that influence tumour evolution [220,221,222]. Through the release of soluble factors, CAFs are able to promote EMT in cancer cells, endowing them with pro-invasive features and stem-like properties, ultimately favouring metastatic dissemination [223,224,225]. CAFs are also responsible for the arising of a pro-inflammatory milieu, for new vessels formation and for the recruitment of immune cells. In particular, CAFs exert an immunomodulating role, mainly enhancing the M2/M1 macrophage and the Treg/Th1 ratio [226,227,228]. Tumour-associated pericytes (TAPs), in addition to their supportive role to the angiogenic process, also actively contribute to TME immunomodulation through disruption of anti-tumour T cells responses [229,230]. In some contexts, the presence of cancer-associated adipocytes (CAAs) is an additional support to tumour development. They represent a lipid reservoir for cancer cells, sustaining their energetic demands [231]. In addition, CAAs exhibit an impairment in adipocyte differentiation markers and over-expression of cytokines, proteases and growth factor termed adipokines, altogether emerging as key actors in neoplastic progression [232,233,234].

## 3. The Involvement of the Endocannabinoid System in the Tumour Microenvironment

Alongside the widely investigated effect of cannabinoid on cancer cells, as deeply aforementioned, evidence are now emerging about the direct effect of ECS on accessory cells of the TME.

The different cellular components of the TME surrounded the tumour as a dense neuralgic network, generating intense crosstalk that supports and promotes neoplastic growth, and they act as allies in all phases of cancer progression, up to the metastatic process. Given the wide effects of ECS, as discussed above, the deregulation of different components of the ECS machinery in the accessory cells may cover an important role in shaping TME.

The microenvironmental cells that are most affected by ECS are the components of the immune system. Immune cells (innate and adaptative immunity) are known to release eCBs and they are also able to respond to these ligands [235,236]. Indeed, CBR expression has been found in several immune components, among which dendritic cells, T- and B-cells, macrophages, and NK cells [237,238,239,240,241]. CB1R and CB2R are mainly expressed in the immune system and their levels are increased upon exposure to inflammatory cytokines (e.g., IL-6, TNF-α) [242]. These cytokines are common in cancer diseases and associated with improvement of cell growth [243,244,245]. The inflammatory milieu also mediates the modulation of eCBs [242]. It is known that cytokines stimulation is able to increase the CBRs expression in peripheral blood mononuclear cells (PBMCs) [246]. However, Staiano et al. reported that the high expression of CB1R and CB2R in lung-resident macrophages inhibited the release of several factors (VEGF-A) in vitro inflammatory environments [247].

Notably, the effects of CBs in tumour immune regulation can be different and dependent on tumour types. In murine TNBC cells, the treatment with CBD reduced the recruitment of the M2 sub-population of macrophages in the primary tumour and in the metastatic site. Indeed, CBD-treated cancer cells exhibited a reduction in secreted granulocyte macrophage colony-stimulation factor (GM-CSF) and C-C motif chemokine ligand 3 (CCL3) cytokines, which are important for macrophage recruitment and activation [142]. Similarly, in melanoma cells THC treatment reduced infiltration of macrophages and neutrophils and interfered with cytokines production [248].

Recent evidence highlighted a central role of CB2R in regulating tumour immunity in melanoma. High intra-tumoural CB2R gene expression correlated to the improvement of overall survival. In particular, CB2R is predominantly expressed in B cells and responsible for their differentiation. The impairment of CB2R expression led to less differentiated B cells, favouring the induction of regulatory T cells (Treg) and the generation of an immunosuppressive microenvironment in *Cnr2^−/−^* mice [249].

In NSCLC, the administration of the CB2R agonist JWH-015 strongly reduced recruitment of macrophages to the tumour site, thereby inhibiting macrophage induced EMT and the acquisition of pro-invasive skills and contributing to the blockade of tumour progression given by the interplay between cancer and microenvironment host cells, thus indicating tumour regressive property in A549, CALU-1 cells and in vivo mouse model [250]. Recently, Haustein and co-workers showed that Met-F-AEA treatment of lung cancer cells led to ICAM-1 upregulation and increased their susceptibility to the cytolytic action of LAK cells, suggesting a novel anti-cancer action mode of cannabinoids [202].

Several studies demonstrated that human DCs expressed CBRs and produced eCBs [12,251]. In the pancreatic ductal adenocarcinoma murine model, 2-AG administration exhibited a direct anti-tumour effect by inducing DC phenotypic maturation and the production of pro-inflammatory cytokines, but also significantly promoted an immunosuppressive microenvironment via increasing the suppressive immune cell population of myeloid-derived suppressor cells (MDSCs) [252].

In HCC, inactivation of CB2R altered immune infiltrates, for instance, leading to inhibition of CD4+ T-cell-recruitment. Although the authors did not investigate the specific lymphocyte population, it seems that the increase in tumour growth in CB2R-deficient HCC model was related to a malfunctioning response in immunosurveillance [253].

In CRC, the GPR55 knockout in mouse models resulted in the alteration of immune composition, with an increase in the amount of CD4+/CD8+ T cells, indicating a relation between GPR55 impairment and a more favourable prognosis [150].

In a model of colon cancer, MAGL deficiency drives CB2R-TLR4 axis-dependent macrophages polarisation towards an M2-phenotype, through 2-AG-CB2R signalling, which contributed to suppressing cancer-related CD8+ T-cells. In mice bearing MAGL-deficient macrophages, treatment with CB2R antagonists created a hurdle to cancer progression, providing potential therapeutic targets [254].

On the contrary, in glioblastoma stem cells (GSCs) ARS2/MAGL axis promoted cancer progression and M2-like TAM polarisation. Furthermore, pharmacological targeting of MAGL impacted on survival rate in vivo xenograft model and offered benefits in patients with glioblastoma multiforme (GBM) [255].

Moreover, cannabinoids have been shown to modulate chemotaxis in various immune cell types [256,257,258]. In line with this concept, THC leads to a CB2R-dependent inhibition of peritoneal murine macrophages through activation of RANTES/CCL5 signalling [259]. A parallel report, by other authors, demonstrated that THC treatment of murine macrophages inhibited their chemotaxis towards CCL2 [260].

In solid tumours, CAFs are one of the most abundant cellular components of the TME. A recent publication shed new light on an intriguing role of the ECS in regulating stromal reactivity in a prostate cancer cell model. Indeed, it was shown that patient-derived CAFs, in response to tumour-secreted inflammatory cytokines, upregulated both CB1R and CB2R compared to the healthy counterpart. Interestingly, treatment with WIN 55,212.2 was able to revert CAF activated phenotype or to prevent tumour-induced healthy prostate fibroblast activation, thereby interfering with the supportive role of the stromal component in prostate cancer [261]. These observations reinforce the therapeutic potential of WIN 55,212-2 in prostate cancer treatment and stress the importance of targeting the endocannabinoid system to simultaneously hinder both cancer cells and stromal compartments and to disrupt their pro-aggressive interplay.

Although in a non-cancer model, it has been reported that UVA-UVB exposition of human skin fibroblast increased CB1R/2 and GPR55 expression, resulting in a pro-inflammatory and pro-oxidant response [262]. This could suggest new approaches to interfere with the stromal ECS in order to prevent UV-induced inflammation and/or redox imbalance. The alteration of CB1R expression was also reported during differentiation of human fibroblast into neurons. Although CB1R was not detected in the early stages of neurogenesis, it was markedly increased during transformation of pluripotent stem cells into differentiated neurons [263].

A few evidence are also available about the impact of cannabinoids on ECs and the angiogenic process in cancer. It was reported that CBD is able to inhibit human umbilical vein endothelial cells (HUVEC) migration, invasion, and sprouting, and that these effects are related to a negative modulation of several prominent factors including MMP-9, TIMP1, PAI1, urokinase-type plasminogen activator (uPA), CXCL16, IL-8, Endothelin-1 (ET-1), and platelet derived growth factor-AA (PDGF-AA), which are involved in the primary vascular endothelial cell functions [264]. In addition, AEA-treated breast cancer cells were found to impair endothelial cell proliferation, which correlated with a decreased synthesis of VEFG, leptin, interferon-γ and thrombopoietin [265]. The anti-angiogenic activity was also demonstrated in thyroid cancer cells, using Met-F-AEA. In particular, this compound reduced endothelial cells proliferation and led to apoptotic process via CB1R. [266].

Recently, Luo and colleagues showed that THC is implicated in the crosstalk between cancer cells and vascular endothelial cells in a Stat1-dependent manner. In particular, the authors demonstrated that THC-treated HCCT116 cells were able to promote tube formation and human-induced pluripotent stem cell-derived vascular endothelial cells (hiPSC-VECs) migration. The genetic and pharmacological Stat1 inhibition interfered with these pro-angiogenic effects, dampening the crosstalk between cancer and endothelial cells [267].

Experimental evidence also demonstrated a role for CBRs activation in mediating cancer cells extravasation and, in turn, metastatic dissemination. In particular, it was reported that CB2R activation with JWH-133 significantly interferes with human melanoma cells adhesion to brain endothelial cells, thereby decreasing the trans-endothelial migration ability and reducing brain melanoma metastases [117].

Additionally, recent studies reported that, although in a non-neoplastic condition, the endogenous 2-AG and AEA, as well as the exogenous cannabinoid WIN 55,212-2, promoted the vasorelaxation of rat retinal capillaries in a CB1R-dependent manner, by modulating the nitric oxide-cyclic guanosine monophosphate pathway, thus resulting in an increase in capillary diameter and pericyte width [268]. The ability of cannabinoids to dilate the retinal capillaries may have therapeutic implications for retinal vascular diseases, and the CB1R may provide a new target for the regulation of the retinal blood flow.

ECS was also involved as a mandatory role in fat storage through different processes, including direct effects on adipocytes (e.g., proliferation and differentiation), contributing to the development of obesity and metabolic diseases [269,270,271]. The presence of CAAs in the tumour *milieu*, of several solid cancers (e.g., in breast cancer) is associated with cancer malignancy and it may be supported by the catabolic effects of ECS. Two different studies reported that the alteration of the catabolic activity in adipocytes led to tumour progression through PPARγ downregulation in co-cultivated adipocytes [272,273]. Moreover, the authors discovered that breast cancer cells co-cultivated with mature adipocytes exhibited an aggressive phenotype, leading to induction of EMT in co-culture condition [273].

**Figure 4:**
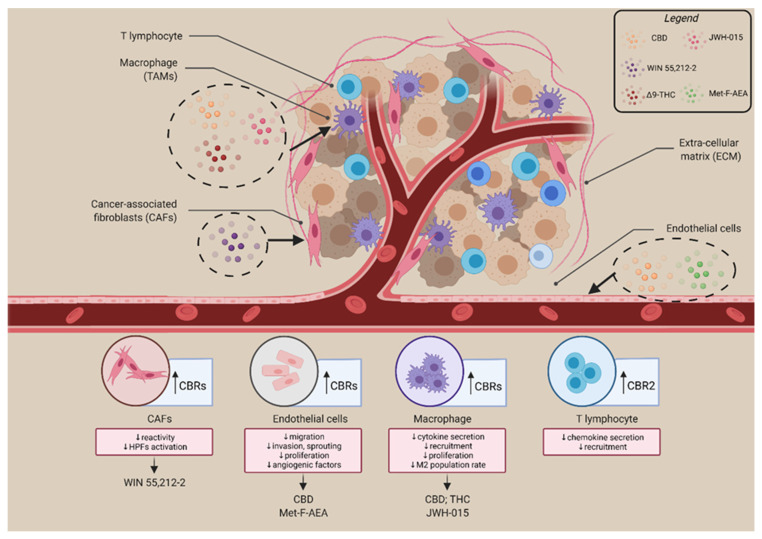
ECS re-shape TME by regulating the functionality and reactivity of different cellular components. Several TME cells express receptors of the ECS; particularly, immune cells, CAFs, and endothelial cells. Cannabinoids are able to mediate several anticancer mechanisms; these bioactive lipids reduce cytokine secretion, T-cell recruitment, proliferation, M2 population rate, thus acting on immune components; reduce CAFs reactivity and invasive ability. On endothelial cells, cannabinoids act on migration, invasion, sprouting features and reduce angiogenic factors release. Cannabinoids showed in the figure: CBD, WIN 55,212-2, Δ9-THC, JWH-015, Met-F-AEA. Created with BioRender.com. Adapted from “Tumor Microenvironment” template, accessed on 16 November 2021.

Although further investigation and clarification are needed, these studies suggest a role for the ECS in regulating the TME composition and reactivity, paving the way to novel pharmacological approaches to fight cancer progression, that not only directly target cancer cells, but also counteract the supportive role of accessory cells (Figure 4).

## 4. Cannabinoid-Based Antineoplastic Treatment—Preclinical Studies

Throughout history, cannabinoids have been considered for their therapeutic features and, in recent decades, they go into the spotlight due to their important role in cancer. However, despite the growing studies to understand the mechanisms by which ECS impacts on cancer progression, there are still few developments in this field that consent to overcome the preclinical phase.

To date, two drugs have been used to fight chemotherapy-induced nausea and vomiting, Dronabinol (Marinol ^®^—Solvay Pharmaceuticals, Inc. Company. Marietta, GA, USA) and Nabilone (Cesamet ^®^—Valeant Pharmaceuticals Int. Costa Mesa, CA, USA), both synthetic forms of Δ9-THC.

Only four pilot clinical studies with cannabinoids in the anti-tumoural therapy were carried out [274,275,276,277]. However, due to the small number of enrolled patients, these studies could not define reliable data on large cohort. Nevertheless, in a future vision, considering the results obtained in vitro and in preclinical models as a firm basis, cannabinoids may potentially be proposed as innovative therapeutic approaches in various cancer types.

In the last decade, several murine preclinical models were set up to test the anti-tumour effect of cannabinoids, as reported in Table 4.

Many authors have explored ECS modulation in preclinical glioma models [26,118,175]. Different studies highlighted that synthetic cannabinoids, such as JWH-133, WIN 55,212-2, HU-210, and KM-233, are promising as anticancer agents for glioma treatment [286]. In particular, the treatment with JWH-133 significantly decreased tumour size and tumour angiogenesis, impairing VEGFR activation and reducing blood vessel size and functionality [282,283]. A deregulated expression of Ang2, TIMP and MMPs given by the synthetic cannabinoid was also observed, resulting in the inhibition of cell invasiveness [286]. Since the upregulation of MMP-2 is associated with poor prognosis in gliomas, the effect on MMP-2 is promising for cannabinoid anti-tumour efficacy [174].

Combined treatment of THC with TMZ, the principal pharmacologic approach for GBM, increased the effect of tumour growth reduction in glioma xenografts. This effect was also observed in tumour resistant to TMZ [278]. CBD also elicited anti-tumour effects, as observed by the decrease in ld-1 and Ki67 expression in tumour both in orthotopic (U251) and xenograft glioma models, significantly prolonging mouse survival [289,290,291]. Moreover, a combination of CBD+THC increased ionising radiation anticancer therapy [175].

Both phytocannabinoids, CBG, and the synthetic HU-331 (quinone, synthesised from CBD) reduced tumour growth in colon cancer xenograft models [279,280]. These treatments also impaired angiogenesis, ACF and gave less toxicity with respect to DOXO [284,292]. CBD was also shown to mainly reduce the number of ACF, polyps and area of tumours in azoxymethane-induced colon cancer, not only impacting on angiogenesis and metastasis, but also promoting apoptotic process mediated by Noxa activation [131], [285]. In addition, the atypical cannabinoid O-1602 exerts an anti-inflammatory effect by inhibiting TNF-α expression and Stat3 and NFkB activation and promoted the decrease in tumour growth in colon cancer murine models [281].

The cannabinoid therapeutic potential was also studied in orthotopic, genetically engineered mouse models (GEMMs) and xenograft breast cancer in vivo models [293]. CBD and THC decreased tumour size and metastatic lung incidence, and in some cases, they additionally increased survival [287]. CBD also acts by decreasing EGFR signal and Akt expression [142]. In HER2+ breast cancer subtypes, THC also impacted the heteromers CB2R-HER2, selectively binding to CBR and consequently leading to HER2 inactivation and decreasing HCC1944-orthotopic xenograft mice tumour size. These findings defined a new targeted approach in HER2-positive breast cancer in in vivo models [90].

Other evidence demonstrated that WIN 55,212-2 significantly impaired tumour growth, angiogenesis, and lung metastasis in two different breast cancer murine models, xenograft-MDA-MB-231 (TNBC subtype) and mouse mammary tumour virus encoding polyomavirus middle T antigen (GEMM-MMTV-PyMT) [119].

In prostate xenograft-LNCaP murine model, CBD was able to induce pro-apoptotic effects and to inhibit tumour growth [134]. On the model, synthetic compounds, such as JWH-015 and WIN 55,212-2, reduced tumour size [98]. In particular, WIN 55,212-2 caused a decrease in serum PSA level that directly correlated with cancer growth inhibition [185].

WIN 55,212-2 was effective also in skin cancers, especially in melanoma, where it reduced tumour growth, angiogenesis and metastatic potential in xenograft-B16 [180]. CBD and THC were also effective in murine models of cutaneous melanoma, leading to a reduction in tumour growth, angiogenesis, and metastatic dissemination, by inducing an increase in autophagy and apoptosis processes [181,248]. In addition, a recent discovery evidenced how the double administration of THC+Trametinib reduces cancer survival, invasion and metastatic potential of MEK inhibitor (MEKi)-resistant melanoma cells [288]. In addition, ACEA was able to inhibit, liver colonisation of human melanoma cells into severe immunodeficiency (SCID) mice [106].

There are also anti-cancer in vivo evidence regarding ECS degradative enzymes inhibitors. It has been reported that URB597, an acknowledged FAAH inhibitor, when used in combination with PEA or AEA, reduces skin cancer and CRC progression, respectively [104,108]. In lung cancer, URB597 was effective on the metastatic potential [163].

URB-602 is a MAGL inhibitor, which showed an anticancer effect on CRC growth and angiogenesis [161]. Another MAGL inhibitor, JZL184, exerted its effects mainly attenuating tumour growth and metastatic process in different murine models, among which, prostate cancer, HCC, and breast cancer, where the compound acts mainly by reducing the progression of bone metastasis [157,159,160]. Lastly, JZL184 increased the survival rate in GBM murine model [255].

These scientific findings concerning in vivo model cannabinoids’ efficacy are positive and encourage more in-depth research in this area. Although considering the obstacles to overcome and the aspects to be better explored, it is extremely interesting to deepen the mechanisms through which ECS deregulation, leads to changes in the tumour *milieu* which then impact on cancer evolution.

## 5. Concluding Remarks

Here, we provide an in-depth overview of the complex endocannabinoid system and the numerous and compelling evidence in the tumour field. The effects of ECS deregulation have been studied for decades. Several evidence explain how ECS can impact on cancer development and what are the signalling pathways that are involved in the observed effects. Most of the evidence is related to cancer cells, where ECS affects tumour initiation and progression through different mechanisms. Cannabinoids induce cell death, cell cycle arrest, inhibition of tumour angiogenesis, but they have a substantial impact also on migration and tumour invasion. However, still today many evidence are contradictory, so they need more in-depth analysis. The surrounding microenvironment is another field that has recently been explored in relation to this complex system. Although the studies are still limited and more deep investigations are needed, there are promising evidence on the deregulation of ECS in the immune system and the role of cannabinoids in cancer immune modulation. A direct effect of the ECS in regulating stromal reactivity, tumour:stroma crosstalk and endothelial cell function is also emerging. In detail, the action of phytocannabinoids (CBD, Δ9-THC), but also the treatment with an aminoalkylindoles agonist (JWH-015) on the immune component, decreases the secretion of cytokines/chemokines, recruitment, and proliferation. Furthermore, for the macrophage component, there is a decrease in the M2 population rate. The use of Met-F-AEA is able to reduce the release of angiogenic factors, proliferation, migration, and sprouting in endothelial cells. A similar result was also obtained with CBD. Finally, it is known, from a recent publication, that the cannabimimetic aminoalkylindole WIN 55,212-2 acts on stromal reactivity and reversion of activating-related phenotype.

This shed new light on the potential clinical application of cannabinoids to target not only cancer cells but also the supportive accessory cells and the tumour:stroma interplay. On the basis of the in vitro available studies, numerous preclinical studies in mouse models have been developed to proceed towards the design of novel pharmacological approaches, based on the use of cannabinoids both alone or in combination with already approved drugs.

## Figures and Tables

**Table 1:** Cannabinoid receptors localisation in human organs, function-related and expression in different tumour types.

RECEPTORS	LOCALIZATION	FUNCTION	TUMOUR	REF.
**CB1R**	Central nervous system Peripheral tissues (e.g., liver, heart, skeletal muscle, adipose tissue, gastro-intestinal tract).	Neurotransmitters release Role in memory Motor coordination Emotional processes	Ovarian tumour Digestive tract Hodgkin lymphoma Prostate cancer	[18,19,20,21,22,24,25,26]
**CB2R**	Lymphoid organs Immune cells Nervous system	Anti-inflammatory Immunosuppressive	Breast cancer Pancreatic tumour Thyroid cancer Prostate cancer	[23,25,26,27]
**GPR55**	Brain Spleen Bones Adipose tissue Langerhans islets	Vascular tone Bone turnover Motor coordination Inflammatory pain Neurological disorders Metabolic/immune dysregulation	Glioma Melanoma Breast cancer Pancreatic tumour	[28,29,30,31,32,33,34,35,36,37,38,39,40]
**TRPV1**	Dorsal root neurons Trigeminal Arteriolar smooth muscle cells Bladder urothelium	Thermoregulation Involved in cough Bladder hyperactivity	Brain tumour Pancreatic tumour Breast cancer Prostate cancer Squamous cell carcinoma	[41,42,43,44,45,46,47,48,49,50,51,52]
**PPARα**	Liver Heart Muscles	Involved in fatty acid catabolism Inflammatory processes	Colon cancer Ovarian tumour Breast cancer Prostate cancer	[53,54,55,56]
**PPARγ** **(γ1, γ2, γ3)**	γ1: ubiquitous γ2: adipose tissue γ3: macrophages	Adipocyte formation Insulin sensitivity Inflammation		

**Table 4:** Anticancer cannabinoid effects in murine preclinical models.

EFFECTS	TUMOUR TYPES and MEDIATORS	REF.
**Tumour growth**	Glioma (JWH-133; THC; CBD) Breast cancer (JWH-133; THC; WIN 55,212-2; JZL184) Prostate cancer (CBD; JWH-015; WIN 55,212-2; JZL184) CRC (CBD; JWH-015; URB597; URB-602; HU-331; O-1602) Melanoma (THC; CBD; WIN 55,212-2; URB597) HCC (JZL184)	[119,134,157,159,160,181,185,248,278,279,280,281]
**Angiogenesis**	Glioma (JWH-133) CRC (CBD; URB-602; CBG; HU-331) Melanoma (THC; CBD; WIN 55,212-2) Breast cancer (WIN 55,212-2)	[98,104,108,118,119,131,161,180,181,248,279,282,283,284,285]
**MMPs expr.**	Brain cancer (JWH-133; THC)	[174,286]
**Apoptosis**	CRC (CBD) Prostate cancer (CBD) Melanoma (THC; CBD)	[131,134,181,248]
**Metastatic incidence**	CRC (CBD) Breast cancer (THC; CBD; WIN 55,212-2; JZL184) Melanoma (THC; CBD; WIN 55,212-2; ACEA) Prostate cancer (WIN 55,212-2; JZL184) Lung cancer (URB597) HCC (JZL184)	[106,119,131,157,159,160,163,180,287,288]
**Survival**	Glioma (CBD; JZL184) Breast cancer (CBD) Melanoma (THC)	[101,255,287,288,289,290,291]

## Data Availability

Not applicable.

## References

[1] Schmid P.C., Schwartz K.D., Smith C.N., Krebsbach R.J., Berdyshev E.V., Schmid H.H. (2000). A sensitive endocannabinoid assay. The simultaneous analysis of N-acylethanolamines and 2-monoacylglycerols. Chem. Phys. Lipids.

[2] Di Marzo V., Fontana A., Cadas H., Schinelli S., Cimino G., Schwartz J.-C., Piomelli D. (1994). Formation and inactivation of endogenous cannabinoid anandamide in central neurons. Nature.

[3] Cadas H., Gaillet S., Beltramo M., Venance L., Piomelli D. (1996). Biosynthesis of an Endogenous Cannabinoid Precursor in Neurons and its Control by Calcium and cAMP. J. Neurosci..

[4] Stella N., Schweitzer P.J., Piomelli D. (1997). A second endogenous cannabinoid that modulates long-term potentiation. Nature.

[5] Bisogno T., Berrendero F., Ambrosino G., Cebeira M., Ramos J., Fernandez-Ruiz J., Di Marzo V. (1999). Brain Regional Distribution of Endocannabinoids: Implications for Their Biosynthesis and Biological Function. Biochem. Biophys. Res. Commun..

[6] Bisogno T., Howell F., Williams G., Minassi A., Cascio M.G., Ligresti A., Matias I., Schiano-Moriello A., Paul P., Williams E.-J. (2003). Cloning of the first sn1-DAG lipases points to the spatial and temporal regulation of endocannabinoid signaling in the brain. J. Cell Biol..

[7] Battista N., Di Tommaso M., Bari M., Maccarrone M. (2012). The endocannabinoid system: An overview. Front. Behav. Neurosci..

[8] Rouzer C.A., Marnett L.J. (2011). Endocannabinoid Oxygenation by Cyclooxygenases, Lipoxygenases, and Cytochromes P450: Cross-Talk between the Eicosanoid and Endocannabinoid Signaling Pathways. Chem. Rev..

[9] Valdeolivas S., Pazos M.R., Bisogno T., Piscitelli F., Iannotti F.A., Allarà M., Sagredo O., Di Marzo V., Fernández-Ruiz J. (2013). The inhibition of 2-arachidonoyl-glycerol (2-AG) biosynthesis, rather than enhancing striatal damage, protects striatal neurons from malonate-induced death: A potential role of cyclooxygenase-2-dependent metabolism of 2-AG. Cell Death Dis..

[10] Weber A., Ni J., Ling K.-H.J., Acheampong A., Tang-Liu D.D.-S., Burk R., Cravatt B.F., Woodward D. (2004). Formation of prostamides from anandamide in FAAH knockout mice analyzed by HPLC with tandem mass spectrometry. J. Lipid Res..

[11] Hillard C.J. (2015). The Endocannabinoid Signaling System in the CNS. Int. Rev. Neurobiol..

[12] Almogi-Hazan O., Or R. (2020). *Cannabis*, the Endocannabinoid System and Immunity—the Journey from the Bedside to the Bench and Back. Int. J. Mol. Sci..

[13] Silvestri C., Di Marzo V. (2013). The Endocannabinoid System in Energy Homeostasis and the Etiopathology of Metabolic Disorders. Cell Metab..

[14] Maurya N., Velmurugan B.K. (2018). Therapeutic applications of cannabinoids. Chem. Interact..

[15] Fraguas-Sánchez A.I., Martín-Sabroso C., Torres-Suárez A.I. (2018). Insights into the effects of the endocannabinoid system in cancer: A review. Br. J. Pharmacol..

[16] Pertwee R.G., Howlett A.C., Abood M.E., Alexander S.P., Di Marzo V., Elphick M.R., Greasley P.J., Hansen H.S., Kunos G., Mackie K. (2010). International Union of Basic and Clinical Pharmacology. LXXIX. Cannabinoid Receptors and Their Ligands: Beyond CB1and CB2. Pharmacol. Rev..

[17] Sharma C., Sadek B., Goyal S.N., Sinha S., Kamal M.A., Ojha S. (2015). Small Molecules from Nature Targeting G-Protein Coupled Cannabinoid Receptors: Potential Leads for Drug Discovery and Development. Evid.-Based Complement. Altern. Med..

[18] Schlicker E., Kathmann M. (2001). Modulation of transmitter release via presynaptic cannabinoid receptors. Trends Pharmacol. Sci..

[19] Herkenham M., Lynn A.B., Little M.D., Johnson M.R., Melvin L.S., de Costa B.R., Rice K.C. (1990). Cannabinoid receptor localization in brain. Proc. Natl. Acad. Sci. USA.

[20] Strangman N.M., Patrick S.L., Hohmann A.G., Tsou K., Walker J. (1998). Evidence for a role of endogenous cannabinoids in the modulation of acute and tonic pain sensitivity. Brain Res..

[21] Matias I., Di Marzo V. (2007). Endocannabinoids and the control of energy balance. Trends Endocrinol. Metab..

[22] Bonz A., Laser M., Küllmer S., Kniesch S., Babin-Ebell J., Popp V., Ertl G., Wagner J.A. (2003). Cannabinoids Acting on CB1 Receptors Decrease Contractile Performance in Human Atrial Muscle. J. Cardiovasc. Pharmacol..

[23] Viscomi M.T., Oddi S., Latini L., Pasquariello N., Florenzano F., Bernardi G., Molinari M., Maccarrone M. (2009). Selective CB2 Receptor Agonism Protects Central Neurons from Remote Axotomy-Induced Apoptosis through the PI3K/Akt Pathway. J. Neurosci..

[24] Pyszniak M., Tabarkiewicz J., Łuszczki J.J. (2016). Endocannabinoid system as a regulator of tumor cell malignancy—biological pathways and clinical significance. OncoTargets Ther..

[25] Preet A., Qamri Z., Nasser M.W., Prasad A., Shilo K., Zou X., Groopman J.E., Ganju R.K. (2010). Cannabinoid Receptors, CB1 and CB2, as Novel Targets for Inhibition of Non–Small Cell Lung Cancer Growth and Metastasis. Cancer Prev. Res..

[26] Pagano C., Navarra G., Coppola L., Bifulco M., Laezza C. (2021). Molecular Mechanism of Cannabinoids in Cancer Progression. Int. J. Mol. Sci..

[27] Laezza C., Pagano C., Navarra G., Pastorino O., Proto M.C., Fiore D., Piscopo C., Gazzerro P., Bifulco M. (2020). The Endocannabinoid System: A Target for Cancer Treatment. Int. J. Mol. Sci..

[28] Lauckner J.E., Jensen J., Chen H.-Y., Lu H.-C., Hille B., Mackie K. (2008). GPR55 is a cannabinoid receptor that increases intracellular calcium and inhibits M current. Proc. Natl. Acad. Sci. USA.

[29] Falasca M., Ferro R. (2016). Role of the lysophosphatidylinositol/GPR55 axis in cancer. Adv. Biol. Regul..

[30] Henstridge C.M., Balenga N.A., Schröder R., Kargl J.K., Platzer W., Martini L., Arthur S., Penman J., Whistler J.L., Kostenis E. (2010). GPR55 ligands promote receptor coupling to multiple signalling pathways. Br. J. Pharmacol..

[31] AlSuleimani Y.M., Hiley C.R. (2015). The GPR55 agonist lysophosphatidylinositol relaxes rat mesenteric resistance artery and induces Ca2+ release in rat mesenteric artery endothelial cells. Br. J. Pharmacol..

[32] Whyte L.S., Ryberg E., Sims N.A., Ridge S.A., Mackie K., Greasley P.J., Ross R.A., Rogers M.J. (2009). The putative cannabinoid receptor GPR55 affects osteoclast function in vitro and bone mass in vivo. Proc Natl Acad Sci USA.

[33] Wu C.S., Chen H., Sun H., Zhu J., Jew C.P., Wager-Miller J., Straiker A., Spencer C., Bradshaw H., Mackie K. (2013). GPR55, a G-Protein Coupled Receptor for Lysophosphatidylinositol, Plays a Role in Motor Coordination. PLoS ONE.

[34] Staton P.C., Hatcher J.P., Walker D.J., Morrison A.D., Shapland E.M., Hughes J.P., Chong E., Mander P.K., Green P.J., Billinton A. (2008). The putative cannabinoid receptor GPR55 plays a role in mechanical hyperalgesia associated with inflammatory and neuropathic pain. Pain.

[35] Guerrero-Alba R., Barragan-Iglesias P., González-Hernández A., Valdez-Moráles E.E., Granados-Soto V., Condés-Lara M., Rodríguez M.G., Marichal-Cancino B.A. (2019). Some Prospective Alternatives for Treating Pain: The Endocannabinoid System and Its Putative Receptors GPR18 and GPR55. Front. Pharmacol..

[36] Tudurí E., Imbernon M., Bautista R.J.H., Tojo M., Fernø J., Diéguez C., Nogueiras R. (2017). GPR55: A new promising target for metabolism?. J. Mol. Endocrinol..

[37] Ford L.A., Roelofs A.J., Anavi-Goffer S., Mowat L., Simpson D.G., Irving A.J., Rogers M.J., Rajnicek A.M., Ross R.A. (2010). A role for L-α-lysophosphatidylinositol and GPR55 in the modulation of migration, orientation and polarization of human breast cancer cells. Br. J. Pharmacol..

[38] Andradas C., Caffarel M.M., Pérez-Gómez E., Salazar M., Lorente M., Velasco G., Guzmán M., Sánchez C. (2011). The orphan G protein-coupled receptor GPR55 promotes cancer cell proliferation via ERK. Oncogene.

[39] Piñeiro R., Maffucci T., Falasca M. (2010). The putative cannabinoid receptor GPR55 defines a novel autocrine loop in cancer cell proliferation. Oncogene.

[40] Perez-Gomez E., Andradas C., Flores J.M., Quintanilla M., Paramio J.M., Guzmán M., Sanchez C. (2012). The orphan receptor GPR55 drives skin carcinogenesis and is upregulated in human squamous cell carcinomas. Oncogene.

[41] Li L., Chen C., Chiang C., Xiao T., Chen Y., Zhao Y., Zheng D. (2021). The Impact of TRPV1 on Cancer Pathogenesis and Therapy: A Systematic Review. Int. J. Biol. Sci..

[42] Fischer M.J.M., Ciotu C.I., Szallasi A. (2020). The Mysteries of Capsaicin-Sensitive Afferents. Front. Physiol..

[43] Caterina M.J., Schumacher M.A., Tominaga M., Rosen T.A., Levine J.D., Julius D. (1997). The capsaicin receptor: A heat-activated ion channel in the pain pathway. Nature.

[44] Helliwell R.J., McLatchie L.M., Clarke M., Winter J., Bevan S., McIntyre P. (1998). Capsaicin sensitivity is associated with the expression of the vanilloid (capsaicin) receptor (VR1) mRNA in adult rat sensory ganglia. Neurosci. Lett..

[45] Kark T., Bagi Z., Lizanecz E., Pásztor E.T., Erdei N., Czikora A., Papp Z., Edes I., Porszasz R., Toth A. (2008). Tissue-Specific Regulation of Microvascular Diameter: Opposite Functional Roles of Neuronal and Smooth Muscle Located Vanilloid Receptor-1. Mol. Pharmacol..

[46] Cavanaugh D.J., Chesler A.T., Jackson A.C., Sigal Y.M., Yamanaka H., Grant R., O’Donnell D., Nicoll R.A., Shah N.M., Julius D. (2011). Trpv1 Reporter Mice Reveal Highly Restricted Brain Distribution and Functional Expression in Arteriolar Smooth Muscle Cells. J. Neurosci..

[47] Birder L., Nakamura Y., Kiss S., Nealen M., Barrick S., Kanai A., Wang E., Ruiz G., De Groat W., Apodaca G. (2002). Altered urinary bladder function in mice lacking the vanilloid receptor TRPV1. Nat. Neurosci..

[48] Tominaga M., Caterina M.J., Malmberg A.B., Rosen T.A., Gilbert H., Skinner K., Raumann B.E., Basbaum A.I., Julius D. (1998). The Cloned Capsaicin Receptor Integrates Multiple Pain-Producing Stimuli. Neuron.

[49] Hartel M., di Mola F.F., Selvaggi F., Mascetta G., Wente M.N., Felix K., Giese N.A., Hinz U., di Sebastiano P., Büchler M.W. (2006). Vanilloids in pancreatic cancer: Potential for chemotherapy and pain management. Gut.

[50] Marincsák R., Tóth B.I., Czifra G., Márton I., Redl P., Tar I., Tóth L., Kovács L., Bíró T. (2009). Increased expression of TRPV1 in squamous cell carcinoma of the human tongue. Oral Dis..

[51] Weber L.V., Al-Refae K., Wölk G., Bonatz G., Altmüller J., Becker C., Gisselmann G., Hatt H. (2016). Expression and functionality of TRPV1 in breast cancer cells. Breast Cancer Targets Ther..

[52] Stock K., Kumar J., Synowitz M., Petrosino S., Imperatore R., Smith E.S.J., Wend P., Purfürst B., Nuber U.A., Gurok U. (2012). Neural precursor cells induce cell death of high-grade astrocytomas through stimulation of TRPV1. Nat. Med..

[53] Stienstra R., Duval C., Muller M., Kersten S. (2006). PPARs, Obesity, and Inflammation. PPAR Res..

[54] Auboeuf D., Rieusset J., Fajas L., Vallier P., Frering V., Riou J.P., Staels B., Auwerx J., Laville M., Vidal H. (1997). Tissue Distribution and Quantification of the Expression of mRNAs of Peroxisome Proliferator-Activated Receptors and Liver X Receptor- in Humans: No Alteration in Adipose Tissue of Obese and NIDDM Patients. Diabetes.

[55] Zhou J., Zhang S., Xue J., Avery J., Wu J., Lind S.E., Ding W.-Q. (2012). Activation of Peroxisome Proliferator-activated Receptor α (PPARα) Suppresses Hypoxia-inducible Factor-1α (HIF-1α) Signaling in Cancer Cells. J. Biol. Chem..

[56] Shigeto T., Yokoyama Y., Xin B., Mizunuma H. (2007). Peroxisome proliferator-activated receptor α and γ ligands inhibit the growth of human ovarian cancer. Oncol. Rep..

[57] Elbegdorj O., Westkaemper R.B., Zhang Y. (2013). A homology modeling study toward the understanding of three-dimensional structure and putative pharmacological profile of the G-protein coupled receptor GPR55. J. Mol. Graph. Model..

[58] Henstridge C.M., Balenga N., Kargl J., Andradas C., Brown A., Irving A., Sanchez C., Waldhoer M. (2011). Minireview: Recent Developments in the Physiology and Pathology of the Lysophosphatidylinositol-Sensitive Receptor GPR55. Mol. Endocrinol..

[59] Alavi M.S., Shamsizadeh A., Azhdari-Zarmehri H., Roohbakhsh A. (2018). Orphan G protein-coupled receptors: The role in CNS disorders. Biomed. Pharmacother..

[60] Pecze L., Blum W., Schwaller B. (2013). Mechanism of capsaicin receptor TRPV1-mediated toxicity in pain-sensing neurons focusing on the effects of Na^+^/Ca^2+^ fluxes and the Ca2+-binding protein calretinin. Biochim. Biophys. Acta Bioenerg..

[61] Romanovsky A.A., Almeida M.C., Garami A., Steiner A., Norman M.H., Morrison S.F., Nakamura K., Burmeister J.J., Nucci T.B. (2009). The Transient Receptor Potential Vanilloid-1 Channel in Thermoregulation: A Thermosensor It Is Not. Pharmacol. Rev..

[62] Cortright D.N., Krause J.E., Broom D.C. (2007). TRP channels and pain. Biochim. Biophys. Acta Mol. Basis Dis..

[63] Adcock J.J. (2009). TRPV1 receptors in sensitisation of cough and pain reflexes. Pulm. Pharmacol. Ther..

[64] Pertwee R.G. (2001). Cannabinoids and the gastrointestinal tract. Gut.

[65] Benz A.H., Renné C., Maronde E., Koch M., Grabiec U., Kallendrusch S., Rengstl B., Newrzela S., Hartmann S., Hansmann M.-L. (2013). Expression and Functional Relevance of Cannabinoid Receptor 1 in Hodgkin Lymphoma. PLoS ONE.

[66] Perez-Gomez E., Andradas C., Blasco-Benito S., Caffarel M.M., García-Taboada E., Villa-Morales M.C., Moreno E., Hamann S., Martin-Villar E., Flores J.M. (2015). Role of Cannabinoid Receptor CB2 in HER2 Pro-oncogenic Signaling in Breast Cancer. J. Natl. Cancer Inst..

[67] Fonseca B.M., Teixeira N.A., Correia-Da-Silva G. (2017). Cannabinoids as Modulators of Cell Death: Clinical Applications and Future Directions. Rev. Physiol. Biochem. Pharmacol..

[68] Javid F.A., Phillips R.M., Afshinjavid S., Verde R., Ligresti A. (2016). Cannabinoid pharmacology in cancer research: A new hope for cancer patients?. Eur. J. Pharmacol..

[69] Vara D., Salazar M., Olea-Herrero N., Guzmán M., Velasco G., Díaz-Laviada I. (2011). Anti-tumoral action of cannabinoids on hepatocellular carcinoma: Role of AMPK-dependent activation of autophagy. Cell Death Differ..

[70] Ferro R., Adamska A., Lattanzio R., Mavrommati I., Edling C.E., Arifin S.A., Fyffe C.A., Sala G., Sacchetto L., Chiorino G. (2018). GPR55 signalling promotes proliferation of pancreatic cancer cells and tumour growth in mice, and its inhibition increases effects of gemcitabine. Oncogene.

[71] Andradas C., Benito S.B., Castillo-Lluva S., Pilla P.D., Alarcia R.D., Garcia A.J., García-Taboada E., Hernando-Llorente R., Soriano J., Hamann S. (2016). Activation of the orphan receptor GPR55 by lysophosphatidylinositol promotes metastasis in triple-negative breast cancer. Oncotarget.

[72] Zhou X.-L., Guo X., Song Y.-P., Zhu C.-Y., Zou W. (2017). The LPI/GPR55 axis enhances human breast cancer cell migration via HBXIP and p-MLC signaling. Acta Pharmacol. Sin..

[73] Adinolfi B., Romanini A., Vanni A., Martinotti E., Chicca A., Fogli S., Nieri P. (2013). Anticancer activity of anandamide in human cutaneous melanoma cells. Eur. J. Pharmacol..

[74] Mikoshiba K. (2015). Role of IP3 receptor signaling in cell functions and diseases. Adv. Biol. Regul..

[75] Oka S., Kimura S., Toshida T., Ota R., Yamashita A., Sugiura T. (2010). Lysophosphatidylinositol induces rapid phosphorylation of p38 mitogen-activated protein kinase and activating transcription factor 2 in HEK293 cells expressing GPR55 and IM-9 lymphoblastoid cells. J. Biochem..

[76] Czifra G., Varga A., Nyeste K., Marincsák R., Tóth B.I., Kovács I., Kovács L., Bíró T. (2008). Increased expressions of cannabinoid receptor-1 and transient receptor potential vanilloid-1 in human prostate carcinoma. J. Cancer Res. Clin. Oncol..

[77] Zhai K., Liskova A., Kubatka P., Büsselberg D. (2020). Calcium Entry through TRPV1: A Potential Target for the Regulation of Proliferation and Apoptosis in Cancerous and Healthy Cells. Int. J. Mol. Sci..

[78] Hu F., Sun W.W., Zhao X.T., Cui Z.J., Yang W.X. (2008). TRPV1 mediates cell death in rat synovial fibroblasts through calcium entry-dependent ROS production and mitochondrial depolarization. Biochem. Biophys. Res. Commun..

[79] Liu T., Wang G., Tao H., Yang Z., Wang Y., Meng Z., Cao R., Xiao Y., Wang X., Zhou J. (2016). Capsaicin mediates caspases activation and induces apoptosis through P38 and JNK MAPK pathways in human renal carcinoma. BMC Cancer.

[80] Amantini C., Mosca M., Nabissi M., Lucciarini R., Caprodossi S., Arcella A., Giangaspero F., Santoni G. (2007). Capsaicin-induced apoptosis of glioma cells is mediated by TRPV1 vanilloid receptor and requires p38 MAPK activation. J. Neurochem..

[81] Ip S.-W., Lan S.-H., Lu H.-F., Huang A.-C., Yang J.-S., Lin J.-P., Huang H.-Y., Lien J.-C., Ho C.-C., Chiu C.-F. (2011). Capsaicin mediates apoptosis in human nasopharyngeal carcinoma NPC-TW 039 cells through mitochondrial depolarization and endoplasmic reticulum stress. Hum. Exp. Toxicol..

[82] Katsuda K., Kataoka M., Uno F., Murakami T., Kondo T., Roth J.A., Tanaka N., Fujiwara T. (2002). Activation of caspase-3 and cleavage of Rb are associated with p16-mediated apoptosis in human non-small cell lung cancer cells. Oncogene.

[83] Hernandez A.M., Colvin E.S., Chen Y.-C., Geiss S.L., Eller L.E., Fueger P.T. (2013). Upregulation of p21 activates the intrinsic apoptotic pathway in β-cells. Am. J. Physiol. Metab..

[84] Cheng E.H.-Y., Kirsch D.G., Clem R.J., Ravi R., Kastan M.B., Bedi A., Ueno K., Hardwick J.M. (1997). Conversion of Bcl-2 to a Bax-like Death Effector by Caspases. Science.

[85] Gil Y.-G., Kang M.-K. (2008). Capsaicin induces apoptosis and terminal differentiation in human glioma A172 cells. Life Sci..

[86] Fiévet C., Fruchart J.-C., Staels B. (2006). PPARα and PPARγ dual agonists for the treatment of type 2 diabetes and the metabolic syndrome. Curr. Opin. Pharmacol..

[87] Moreno E., Andradas C., Medrano M., Caffarel M.M., Perez-Gomez E., Blasco-Benito S., Gómez-Cañas M., Pazos M.R., Irving A.J., Lluís C. (2014). Targeting CB2-GPR55 Receptor Heteromers Modulates Cancer Cell Signaling. J. Biol. Chem..

[88] Coke C.J., Scarlett K.A., Chetram M.A., Jones K.J., Sandifer B.J., Davis A.S., Marcus A.I., Hinton C.V. (2016). Simultaneous Activation of Induced Heterodimerization between CXCR4 Chemokine Receptor and Cannabinoid Receptor 2 (CB2) Reveals a Mechanism for Regulation of Tumor Progression. J. Biol. Chem..

[89] Scarlett K.A., White E.S.Z., Coke C.J., Carter J.R., Bryant L.K., Hinton C.V. (2018). Agonist-induced CXCR4 and CB2 Heterodimerization Inhibits Gα13/RhoA-mediated Migration. Mol. Cancer Res..

[90] Blasco-Benito S., Moreno E., Seijo-Vila M., Tundidor I., Andradas C., Caffarel M.M., Caro-Villalobos M., Urigüen L., Diez-Alarcia R., Moreno-Bueno G. (2019). Therapeutic targeting of HER2–CB2R heteromers in HER2-positive breast cancer. Proc. Natl. Acad. Sci. USA.

[91] Nikan M., Nabavi S.M., Manayi A. (2016). Ligands for cannabinoid receptors, promising anticancer agents. Life Sci..

[92] Pertwee R.G. (2006). Cannabinoid pharmacology: The first 66 years. Br. J. Pharmacol..

[93] Soethoudt M., Grether U., Fingerle J., Grim T.W., Fezza F., De Petrocellis L., Ullmer C., Rothenhäusler B., Perret C., Van Gils N. (2017). Cannabinoid CB2 receptor ligand profiling reveals biased signalling and off-target activity. Nat. Commun..

[94] Ryberg E., Larsson N., Sjögren S., Hjorth S., Hermansson N.-O., Leonova J., Elebring T., Nilsson K., Drmota T., Greasley P.J. (2007). The orphan receptor GPR55 is a novel cannabinoid receptor. Br. J. Pharmacol..

[95] Gavva N.R., Klionsky L., Qu Y., Shi L., Tamir R., Edenson S., Zhang T.J., Viswanadhan V.N., Tóth A., Pearce L.V. (2004). Molecular Determinants of Vanilloid Sensitivity in TRPV1. J. Biol. Chem..

[96] De Petrocellis L., Di Marzo V. (2009). An introduction to the endocannabinoid system: From the early to the latest concepts. Best Pr. Res. Clin. Endocrinol. Metab..

[97] Eichele K., Ramer R., Hinz B. (2008). R(+)-Methanandamide-Induced Apoptosis of Human Cervical Carcinoma Cells Involves A Cyclooxygenase-2-Dependent Pathway. Pharm. Res..

[98] Olea-Herrero N., Vara D., Malagarie-Cazenave S., Diazlaviada I. (2009). Inhibition of human tumour prostate PC-3 cell growth by cannabinoids R(+)-Methanandamide and JWH-015: Involvement of CB2. Br. J. Cancer.

[99] Ortega A., García-Hernández V.M., Ruiz-Garcia E., Meneses-García A., Herrera-Gomez A., Aguilar-Ponce J.L., Montes-Servín E., Prospero-García O., Del Angel S.A. (2016). Comparing the effects of endogenous and synthetic cannabinoid receptor agonists on survival of gastric cancer cells. Life Sci..

[100] Laezza C., Pisanti S., Malfitano A.M., Bifulco M. (2008). The anandamide analog, Met-F-AEA, controls human breast cancer cell migration via the RHOA/RHO kinase signaling pathway. Endocr.-Related Cancer.

[101] Grimaldi C., Pisanti S., Laezza C., Malfitano A.M., Santoro A., Vitale M., Caruso M.G., Notarnicola M., Iacuzzo I., Portella G. (2006). Anandamide inhibits adhesion and migration of breast cancer cells. Exp. Cell Res..

[102] Laezza C., Pisanti S., Crescenzi E., Bifulco M. (2006). Anandamide inhibits Cdk2 and activates Chk1 leading to cell cycle arrest in human breast cancer cells. FEBS Lett..

[103] Ravi J., Sneh A., Shilo K., Nasser M.W., Ganju R.K. (2014). FAAH inhibition enhances anandamide mediated anti-tumorigenic effects in non-small cell lung cancer by downregulating the EGF/EGFR pathway. Oncotarget.

[104] Proto M.C., Gazzerro P., Di Croce L., Santoro A., Malfitano A.M., Pisanti S., Laezza C., Bifulco M. (2011). Interaction of endocannabinoid system and steroid Hormones in the control of colon cancer cell growth. J. Cell. Physiol..

[105] Cozzolino R., Calì G., Bifulco M., Laccetti P. (2009). A metabolically stable analogue of anandamide, Met-F-AEA, inhibits human thyroid carcinoma cell lines by activation of apoptosis. Investig. New Drugs.

[106] Kenessey I., Bánki B., Márk Á., Varga N., Tóvári J., Ladányi A., Rásó E., Tímár J. (2012). Revisiting CB1 Receptor as Drug Target in Human Melanoma. Pathol. Oncol. Res..

[107] Petrosino S., Di Marzo V. (2016). The pharmacology of palmitoylethanolamide and first data on the therapeutic efficacy of some of its new formulations. Br. J. Pharmacol..

[108] Hamtiaux L., Masquelier J., Muccioli G.G., Bouzin C., Feron O., Gallez B., Lambert D.M. (2012). The association of N-palmitoylethanolamine with the FAAH inhibitor URB597 impairs melanoma growth through a supra-additive action. BMC Cancer.

[109] De Petrocellis L., Bisogno T., Ligresti A., Bifulco M., Melck D., Di Marzo V. (2002). Effect on cancer cell proliferation of palmitoylethanolamide, a fatty acid amide interacting with both the cannabinoid and vanilloid signalling systems. Fundam. Clin. Pharmacol..

[110] Cianchi F., Papucci L., Schiavone N., Lulli M., Magnelli L., Vinci M.C., Messerini L., Manera C., Ronconi E., Romagnani P. (2008). Cannabinoid Receptor Activation Induces Apoptosis through Tumor Necrosis Factor α–Mediated Ceramide De novo Synthesis in Colon Cancer Cells. Clin. Cancer Res..

[111] Pourkhalili N., Ghahremani M.H., Farsandaj N., Tavajohi S., Majdzadeh M., Parsa M., Lavasani N.J., Ostad S.N. (2012). Evaluation of anti-invasion effect of cannabinoids on human hepatocarcinoma cells. Toxicol. Mech. Methods.

[112] Mohammadpour F., Ostad S.N., Aliebrahimi S., Daman Z. (2017). Anti-invasion effects of cannabinoids agonist and antagonist on human breast cancer stem cells. Iran. J. Pharm. Res. IJPR.

[113] Dando I., Donadelli M., Costanzo C., Dalla Pozza E., D’Alessandro A., Zolla L., Palmieri M. (2013). Cannabinoids inhibit energetic metabolism and induce AMPK-dependent autophagy in pancreatic cancer cells. Cell Death Dis..

[114] Donadelli M., Dando I., Zaniboni T., Costanzo C., Pozza E.D., Scupoli M., Scarpa A., Zappavigna S., Marra M., Abbruzzese A. (2011). Gemcitabine/cannabinoid combination triggers autophagy in pancreatic cancer cells through a ROS-mediated mechanism. Cell Death Dis..

[115] Nasser M.W., Qamri Z., Deol Y.S., Smith D., Shilo K., Zou X., Ganju R.K. (2011). Crosstalk between Chemokine Receptor CXCR4 and Cannabinoid Receptor CB2 in Modulating Breast Cancer Growth and Invasion. PLoS ONE.

[116] Elbaz M., Ahirwar D., Ravi J., Nasser M.W., Ganju R.K. (2016). Novel role of cannabinoid receptor 2 in inhibiting EGF/EGFR and IGF-I/IGF-IR pathways in breast cancer. Oncotarget.

[117] Haskó J., Fazakas C., Molnár J., Nyúl-Tóth Á., Herman H., Hermenean A., Wilhelm I., Persidsky Y., Krizbai I.A. (2014). CB2 Receptor Activation Inhibits Melanoma Cell Transmigration through the Blood-Brain Barrier. Int. J. Mol. Sci..

[118] Sanchez C., de Ceballos M.L., Gomez del Pulgar T., Rueda D., Corbacho C., Velasco G., Galve-Roperh I., Huffman J.W., y Cajal S.R., Guzmán M. (2001). Inhibition of glioma growth in vivo by selective activation of the CB(2) cannabinoid receptor. Cancer Res..

[119] Qamri Z., Preet A., Nasser M.W., Bass C.E., Leone G., Barsky S.H., Ganju R.K. (2009). Synthetic cannabinoid receptor agonists inhibit tumor growth and metastasis of breast cancer. Mol. Cancer Ther..

[120] McPartland J.M., Duncan M., Di Marzo V., Pertwee R. (2015). Are cannabidiol and Δ9-tetrahydrocannabivarin negative modulators of the endocannabinoid system? A systematic review. Br. J. Pharmacol..

[121] Ożarowski M., Karpiński T., Zielińska A., Souto E., Wielgus K. (2021). Cannabidiol in Neurological and Neoplastic Diseases: Latest Developments on the Molecular Mechanism of Action. Int. J. Mol. Sci..

[122] Sharir H., Abood M.E. (2010). Pharmacological characterization of GPR55, a putative cannabinoid receptor. Pharmacol. Ther..

[123] O’Sullivan S.E. (2016). An update on PPAR activation by cannabinoids. Br. J. Pharmacol..

[124] Anand U., Jones B., Korchev Y., Bloom S.R., Pacchetti B., Anand P., Sodergren M.H. (2020). CBD Effects on TRPV1 Signaling Pathways in Cultured DRG Neurons. J. Pain Res..

[125] Pertwee R.G. (2007). GPR55: A new member of the cannabinoid receptor clan?. Br. J. Pharmacol..

[126] Santoro A., Pisanti S., Grimaldi C., Izzo A., Borrelli F., Proto M.C., Malfitano A.M., Gazzerro P., Laezza C., Bifulco M. (2009). Rimonabant inhibits human colon cancer cell growth and reduces the formation of precancerous lesions in the mouse colon. Int. J. Cancer.

[127] Sarnataro D., Pisanti S., Santoro A., Gazzerro P., Malfitano A.M., Laezza C., Bifulco M. (2006). The Cannabinoid CB1 Receptor Antagonist Rimonabant (SR141716) Inhibits Human Breast Cancer Cell Proliferation through a Lipid Raft-Mediated Mechanism. Mol. Pharmacol..

[128] Ciaglia E., Torelli G., Pisanti S., Picardi P., D’Alessandro A., Laezza C., Malfitano A.M., Fiore D., Zottola A.C.P., Proto M.C. (2015). Cannabinoid receptor CB1 regulates STAT3 activity and its expression dictates the responsiveness to SR141716 treatment in human glioma patients’ cells. Oncotarget.

[129] Fiore D., Ramesh P., Proto M.C., Piscopo C., Franceschelli S., Anzelmo S., Medema J.P., Bifulco M., Gazzerro P. (2018). Rimonabant Kills Colon Cancer Stem Cells without Inducing Toxicity in Normal Colon Organoids. Front. Pharmacol..

[130] Zhang X., Qin Y., Pan Z., Li M., Liu X., Chen X., Qu G., Zhou L., Xu M., Zheng Q. (2019). Cannabidiol Induces Cell Cycle Arrest and Cell Apoptosis in Human Gastric Cancer SGC-7901 Cells. Biomolecules.

[131] Jeong S., Yun H.K., Jeong Y.A., Jo M.J., Kang S.H., Kim J.L., Kim D.Y., Park S.H., Kim B.R., Na Y.J. (2019). Cannabidiol-induced apoptosis is mediated by activation of Noxa in human colorectal cancer cells. Cancer Lett..

[132] Marcu J.P., Christian R.T., Lau D., Zielinski A.J., Horowitz M.P., Lee J., Pakdel A., Allison J., Limbad C., Moore D.H. (2010). Cannabidiol Enhances the Inhibitory Effects of Δ9-Tetrahydrocannabinol on Human Glioblastoma Cell Proliferation and Survival. Mol. Cancer Ther..

[133] Ligresti A., Moriello A.S., Starowicz K., Matias I., Pisanti S., De Petrocellis L., Laezza C., Portella G., Bifulco M., Di Marzo V. (2006). Antitumor Activity of Plant Cannabinoids with Emphasis on the Effect of Cannabidiol on Human Breast Carcinoma. J. Pharmacol. Exp. Ther..

[134] De Petrocellis L., Ligresti A., Moriello A.S., Iappelli M., Verde R., Stott C.G., Cristino L., Orlando P., Di Marzo V. (2012). Non-THC cannabinoids inhibit prostate carcinoma growthin vitroandin vivo: Pro-apoptotic effects and underlying mechanisms. Br. J. Pharmacol..

[135] Ramer R., Heinemann K., Merkord J., Rohde H., Salamon A., Linnebacher M., Hinz B. (2012). COX-2 and PPAR-γ Confer Cannabidiol-Induced Apoptosis of Human Lung Cancer Cells. Mol. Cancer Ther..

[136] Sultan A.S., Marie M., Sheweita S.A. (2018). Novel mechanism of cannabidiol-induced apoptosis in breast cancer cell lines. Breast.

[137] Shrivastava A., Kuzontkoski P.M., Groopman J.E., Prasad A. (2011). Cannabidiol Induces Programmed Cell Death in Breast Cancer Cells by Coordinating the Cross-talk between Apoptosis and Autophagy. Mol. Cancer Ther..

[138] McAllister S.D., Christian R.T., Horowitz M.P., Garcia A., Desprez P.-Y. (2007). Cannabidiol as a novel inhibitor of Id-1 gene expression in aggressive breast cancer cells. Mol. Cancer Ther..

[139] Aviello G., Romano B., Borrelli F., Capasso R., Gallo L., Piscitelli F., Di Marzo V., Izzo A.A. (2012). Chemopreventive effect of the non-psychotropic phytocannabinoid cannabidiol on experimental colon cancer. J. Mol. Med..

[140] Ramer R., Rohde A., Merkord J., Rohde H., Hinz B. (2010). Decrease of Plasminogen Activator Inhibitor-1 May Contribute to the Anti-Invasive Action of Cannabidiol on Human Lung Cancer Cells. Pharm. Res..

[141] Ramer R., Bublitz K., Freimuth N., Merkord J., Rohde H., Haustein M., Borchert P., Schmuhl E., Linnebacher M., Hinz B. (2011). Cannabidiol inhibits lung cancer cell invasion and metastasis via intercellular adhesion molecule-1. FASEB J..

[142] Elbaz M., Nasser M.W., Ravi J., Wani N.A., Ahirwar D.K., Zhao H., Oghumu S., Satoskar A.R., Shilo K., Carson W.E. (2015). Modulation of the tumor microenvironment and inhibition of EGF/EGFR pathway: Novel anti-tumor mechanisms of Cannabidiol in breast cancer. Mol. Oncol..

[143] Solinas M., Massi P., Cinquina V., Valenti M., Bolognini D., Gariboldi M., Monti E., Rubino T., Parolaro D. (2013). Cannabidiol, a Non-Psychoactive Cannabinoid Compound, Inhibits Proliferation and Invasion in U87-MG and T98G Glioma Cells through a Multitarget Effect. PLoS ONE.

[144] Soroceanu L., Murase R., Limbad C., Singer E., Allison J., Adrados I., Kawamura R., Pakdel A., Fukuyo Y., Nguyen D. (2012). Id-1 Is a Key Transcriptional Regulator of Glioblastoma Aggressiveness and a Novel Therapeutic Target. Cancer Res..

[145] Elbaz M., Ahirwar D., Xiaoli Z., Zhou X., Lustberg M., Nasser M.W., Shilo K., Ganju R.K. (2016). TRPV2 is a novel biomarker and therapeutic target in triple negative breast cancer. Oncotarget.

[146] Nabissi M., Morelli M.B., Santoni M., Santoni G. (2012). Triggering of the TRPV2 channel by cannabidiol sensitizes glioblastoma cells to cytotoxic chemotherapeutic agents. Carcinogenesis.

[147] Scott K., Dennis J.L., Dalgleish A.G., Liu W.M. (2015). Inhibiting Heat Shock Proteins Can Potentiate the Cytotoxic Effect of Cannabidiol in Human Glioma Cells. Anticancer. Res..

[148] Wang J., Xu Y., Zou Y., Zhu L., Dong B., Huang J., Chen Y., Xue W., Huang Y., Kong W. (2016). Overexpression of cannabinoid receptor 1 promotes renal cell carcinoma progression. Tumor Biol..

[149] Wang J., Xu Y., Zhu L., Zou Y., Kong W., Dong B., Huang J., Chen Y., Xue W., Huang Y. (2017). Cannabinoid receptor 2 as a novel target for promotion of renal cell carcinoma prognosis and progression. J. Cancer Res. Clin. Oncol..

[150] Hasenoehrl C., Feuersinger D., Sturm E.M., Bärnthaler T., Heitzer E., Graf R., Grill M., Pichler M., Beck S., Butcher L. (2018). G protein-coupled receptor GPR55 promotes colorectal cancer and has opposing effects to cannabinoid receptor 1. Int. J. Cancer.

[151] Kargl J., Andersen L., Hasenöhrl C., Feuersinger D., Stancic A., Fauland A., Magnes C., El-Heliebi A., Lax S., Uranitsch S. (2015). GPR55 promotes migration and adhesion of colon cancer cells indicating a role in metastasis. Br. J. Pharmacol..

[152] Singh N.S., Bernier M., Wainer I.W. (2016). Selective GPR55 antagonism reduces chemoresistance in cancer cells. Pharmacol. Res..

[153] Wu X., Han L., Zhang X., Li L., Jiang C., Qiu Y., Huang R., Xie B., Lin Z., Ren J. (2012). Alteration of endocannabinoid system in human gliomas. J. Neurochem..

[154] Ayakannu T., Taylor A.H., Bari M., Mastrangelo N., Maccarrone M., Konje J.C. (2019). Expression and Function of the Endocannabinoid Modulating Enzymes Fatty Acid Amide Hydrolase and N-Acylphosphatidylethanolamine-Specific Phospholipase D in Endometrial Carcinoma. Front. Oncol..

[155] Endsley M.P., Thill R., Choudhry I., Williams C.L., Kajdacsy-Balla A., Campbell W.B., Nithipatikom K. (2008). Expression and function of fatty acid amide hydrolase in prostate cancer. Int. J. Cancer.

[156] Zhu W., Zhao Y., Zhou J., Wang X., Pan Q., Zhang N., Wang L., Wang M., Zhan D., Liu Z. (2016). Monoacylglycerol lipase promotes progression of hepatocellular carcinoma via NF-κB-mediated epithelial-mesenchymal transition. J. Hematol. Oncol..

[157] Nomura D.K., Lombardi D.P., Chang J.W., Niessen S., Ward A.M., Long J.Z., Hoover H.H., Cravatt B.F. (2011). Monoacylglycerol Lipase Exerts Dual Control over Endocannabinoid and Fatty Acid Pathways to Support Prostate Cancer. Chem. Biol..

[158] Ma M., Bai J., Ling Y., Chang W., Xie G., Li R., Wang G., Tao K. (2016). Monoacylglycerol lipase inhibitor JZL184 regulates apoptosis and migration of colorectal cancer cells. Mol. Med. Rep..

[159] Zhang J., Liu Z., Lian Z., Liao R., Chen Y., Qin Y., Wang J., Jiang Q., Wang X., Gong J. (2016). Monoacylglycerol Lipase: A Novel Potential Therapeutic Target and Prognostic Indicator for Hepatocellular Carcinoma. Sci. Rep..

[160] Marino S., de Ridder D., Bishop R.T., Renema N., Ponzetti M., Sophocleous A., Capulli M., Aljeffery A., Carrasco G., Gens M.D. (2019). Paradoxical effects of JZL184, an inhibitor of monoacylglycerol lipase, on bone remodelling in healthy and cancer-bearing mice. EBioMedicine.

[161] Pagano E., Borrelli F., Orlando P., Romano B., Monti M., Morbidelli L., Aviello G., Imperatore R., Capasso R., Piscitelli F. (2017). Pharmacological inhibition of MAGL attenuates experimental colon carcinogenesis. Pharmacol. Res..

[162] Orrego-González E., Londoño-Tobón L., Ardila-González J., Polania-Tovar D., Valencia-Cárdenas A., Meerbeke A.V.-V. (2020). Cannabinoid Effects on Experimental Colorectal Cancer Models Reduce Aberrant Crypt Foci (ACF) and Tumor Volume: A Systematic Review. Evid.-Based Complement. Altern. Med..

[163] Winkler K., Ramer R., Dithmer S., Ivanov I., Merkord J., Hinz B. (2016). Fatty acid amide hydrolase inhibitors confer anti-invasive and antimetastatic effects on lung cancer cells. Oncotarget.

[164] Greenhough A., Patsos H.A., Williams A.C., Paraskeva C. (2007). The cannabinoid δ9-tetrahydrocannabinol inhibits RAS-MAPK and PI3K-AKT survival signalling and induces BAD-mediated apoptosis in colorectal cancer cells. Int. J. Cancer.

[165] Vara D., Morell C.M., Rodríguez-Henche N., Díaz-Laviada I. (2013). Involvement of PPARγ in the antitumoral action of cannabinoids on hepatocellular carcinoma. Cell Death Dis..

[166] Munson A.E., Harris L.S., Friedman M.A., Dewey W.L., Carchman R.A. (1975). Antineoplastic Activity of Cannabinoids2. J. Natl. Cancer Inst..

[167] Preet A., Ganju R.K., E Groopman J. (2007). Δ9-Tetrahydrocannabinol inhibits epithelial growth factor-induced lung cancer cell migration in vitro as well as its growth and metastasis in vivo. Oncogene.

[168] Caffarel M.M., Sarrió D., Palacios J., Guzmán M., Sanchez C. (2006). Δ9-Tetrahydrocannabinol Inhibits Cell Cycle Progression in Human Breast Cancer Cells through Cdc2 Regulation. Cancer Res..

[169] Von Bueren A.O., Schlumpf M., Lichtensteiger W. (2008). Delta(9)-tetrahydrocannabinol inhibits 17beta-estradiol-induced proliferation and fails to activate androgen and estrogen receptors in MCF7 human breast cancer cells. Anticancer. Res..

[170] Ruiz L., Miguel A., Díaz-Laviada I. (1999). Δ9-Tetrahydrocannabinol induces apoptosis in human prostate PC-3 cells via a receptor-independent mechanism. FEBS Lett..

[171] Carracedo A., Lorente M., Egia A., Blázquez C., García S., Giroux V., Malicet C., Villuendas R., Gironella M., González-Feria L. (2006). The stress-regulated protein p8 mediates cannabinoid-induced apoptosis of tumor cells. Cancer Cell.

[172] Galanti G., Fisher T., Kventsel I., Shoham J., Gallily R., Mechoulam R., Lavie G., Amariglio N., Rechavi G., Toren A. (2008). Δ9-Tetrahydrocannabinol inhibits cell cycle progression by downregulation of E2F1 in human glioblastoma multiforme cells. Acta Oncol..

[173] Hernández-Tiedra S., Fabrias G., Dávila D., Salanueva J., Casas J., Montes L.R., Antón Z., García-Taboada E., Salazar-Roa M., Lorente M. (2016). Dihydroceramide accumulation mediates cytotoxic autophagy of cancer cells via autolysosome destabilization. Autophagy.

[174] Blázquez C., Salazar M., Carracedo A., Lorente M., Egia A., González-Feria L., Haro A., Velasco G., Guzmán M. (2008). Cannabinoids Inhibit Glioma Cell Invasion by Down-regulating Matrix Metalloproteinase-2 Expression. Cancer Res..

[175] Scott K., Dalgleish A.G., Liu W.M. (2014). The Combination of Cannabidiol and Δ9-Tetrahydrocannabinol Enhances the Anticancer Effects of Radiation in an Orthotopic Murine Glioma Model. Mol. Cancer Ther..

[176] Zhang Y., Zheng W., Shen K., Shen W. (2018). ∆9-tetrahydrocannabinol inhibits epithelial-mesenchymal transition and metastasis by targeting matrix metalloproteinase-9 in endometrial cancer. Oncol. Lett..

[177] Powles T., Poele R.T., Shamash J., Chaplin T., Propper D., Joel S., Oliver T., Liu W.M. (2005). Cannabis-induced cytotoxicity in leukemic cell lines: The role of the cannabinoid receptors and the MAPK pathway. Blood.

[178] Holland M., Panetta J., Hoskins J., Bebawy M., Roufogalis B., Allen J., Arnold J. (2006). The effects of cannabinoids on P-glycoprotein transport and expression in multidrug resistant cells. Biochem. Pharmacol..

[179] Scott K.A., Dalgleish A.G., Liu W.M. (2017). Anticancer effects of phytocannabinoids used with chemotherapy in leukaemia cells can be improved by altering the sequence of their administration. Int. J. Oncol..

[180] Blázquez C., Carracedo A., Barrado L., Real P.J., Fernández-Luna J.L., Velasco G., Malumbres M., Guzmán M. (2006). Cannabinoid receptors as novel targets for the treatment of melanoma. FASEB J..

[181] Armstrong J.L., Hill D.S., McKee C.S., Hernandez-Tiedra S., Lorente M., Lopez-Valero I., Anagnostou M.E., Babatunde F., Corazzari M., Redfern C.P.F. (2015). Exploiting Cannabinoid-Induced Cytotoxic Autophagy to Drive Melanoma Cell Death. J. Investig. Dermatol..

[182] Xian X.-S., Park H., Cho Y.K., Lee I.S., Kim S.W., Choi M.-G., Chung I.-S., Han K.-H., Park J.M. (2010). Effect of a synthetic cannabinoid agonist on the proliferation and invasion of gastric cancer cells. J. Cell. Biochem..

[183] Xian X.-S., Park H., Choi M.-G., Park J.M. (2013). Cannabinoid Receptor Agonist as an Alternative Drug in 5-Fluorouracil-Resistant Gastric Cancer Cells. Anticancer Res..

[184] Xian X., Huang L., Zhang B., Wu C., Cui J., Wang Z. (2016). WIN 55,212-2 Inhibits the Epithelial Mesenchymal Transition of Gastric Cancer Cells via COX-2 Signals. Cell. Physiol. Biochem..

[185] Sarfaraz S., Afaq F., Adhami V.M., Mukhtar H. (2005). Cannabinoid Receptor as a Novel Target for the Treatment of Prostate Cancer. Cancer Res..

[186] Morell C., Bort A., Vara-Ciruelos D., Ramos-Torres A., Rodríguez-Henche N., Díaz-Laviada I. (2016). The cannabinoid WIN 55,212-2 prevents neuroendocrine differentiation of LNCaP prostate cancer cells. Prostate Cancer Prostatic Dis..

[187] Khan M.I., Sobocińska A.A., Brodaczewska K.K., Zielniok K., Gajewska M., Kieda C., Czarnecka A.M., Szczylik C. (2018). Involvement of the CB2 cannabinoid receptor in cell growth inhibition and G0/G1 cell cycle arrest via the cannabinoid agonist WIN 55,212–2 in renal cell carcinoma. BMC Cancer.

[188] Notaro A., Emanuele S., Geraci F., D’Anneo A., Lauricella M., Calvaruso G., Giuliano M. (2019). WIN55,212-2-Induced Expression of Mir-29b1 Favours the Suppression of Osteosarcoma Cell Migration in a SPARC-Independent Manner. Int. J. Mol. Sci..

[189] Müller L., Radtke A., Decker J., Koch M., Belge G. (2017). The Synthetic Cannabinoid WIN 55,212-2 Elicits Death in Human Cancer Cell Lines. Anticancer. Res..

[190] DeMorrow S., Francis H., Gaudio E., Venter J., Franchitto A., Kopriva S., Onori P., Mancinelli R., Frampton G., Coufal M. (2008). The endocannabinoid anandamide inhibits cholangiocarcinoma growth via activation of the noncanonical Wnt signaling pathway. Am. J. Physiol. Liver Physiol..

[191] Huang L., Ramirez J.C., Frampton G.A., Golden L.E., Quinn M.A., Pae H.Y., Horvat D., Liang L.-J., DeMorrow S. (2011). Anandamide exerts its antiproliferative actions on cholangiocarcinoma by activation of the GPR55 receptor. Lab. Investig..

[192] Melck D., Rueda D., Galve-Roperh I., De Petrocellis L., Guzmán M., Di Marzo V. (1999). Involvement of the cAMP/protein kinase A pathway and of mitogen-activated protein kinase in the anti-proliferative effects of anandamide in human breast cancer cells. FEBS Lett..

[193] De Petrocellis L., Melck D., Palmisano A., Bisogno T., Laezza C., Bifulco M., Di Marzo V. (1998). The endogenous cannabinoid anandamide inhibits human breast cancer cell proliferation. Proc. Natl. Acad. Sci. USA.

[194] Melck D., De Petrocellis L., Orlando P., Bisogno T., Laezza C., Bifulco M., Di Marzo V. (2000). Suppression of Nerve Growth Factor Trk Receptors and Prolactin Receptors by Endocannabinoids Leads to Inhibition of Human Breast and Prostate Cancer Cell Proliferation. Endocrinology.

[195] Mimeault M., Pommery N., Wattez N., Bailly C., Hénichart J.-P. (2003). Anti-proliferative and apoptotic effects of anandamide in human prostatic cancer cell lines: Implication of epidermal growth factor receptor down-regulation and ceramide production. Prostate.

[196] Orellana-Serradell O., Poblete C.E., Sanchez C., Castellón E.A., Gallegos I., Huidobro C., Llanos M.N., Contreras H.R. (2015). Proapoptotic effect of endocannabinoids in prostate cancer cells. Oncol. Rep..

[197] Soliman E., Van Dross R. (2015). Anandamide-induced endoplasmic reticulum stress and apoptosis are mediated by oxidative stress in non-melanoma skin cancer: Receptor-independent endocannabinoid signaling. Mol. Carcinog..

[198] Flygare J., Gustafsson K., Kimby E., Christensson B., Sander B. (2005). Cannabinoid receptor ligands mediate growth inhibition and cell death in mantle cell lymphoma. FEBS Lett..

[199] Vanderah T.W., Hanlon K.E., Lozano-Ondoua A.N., Umaretiya P.J., Symons-Liguori A.M., Chandramouli A., Moy J.K., Kwass W.K., Mantyh P.W., Nelson M.A. (2016). Modulation of breast cancer cell viability by a cannabinoid receptor 2 agonist, JWH-015, is calcium dependent. Breast Cancer Targets Ther..

[200] Gazzerro P., Malfitano A.M., Proto M.C., Santoro A., Pisanti S., Caruso M.G., Notarnicola M., Messa C., Laezza C., Misso G. (2010). Synergistic inhibition of human colon cancer cell growth by the cannabinoid CB1 receptor antagonist rimonabant and oxaliplatin. Oncol. Rep..

[201] Proto M.C., Fiore D., Piscopo C., Franceschelli S., Bizzarro V., Laezza C., Lauro G., Feoli A., Tosco A., Bifulco G. (2017). Inhibition of Wnt/β-Catenin pathway and Histone acetyltransferase activity by Rimonabant: A therapeutic target for colon cancer. Sci. Rep..

[202] Haustein M., Ramer R., Linnebacher M., Manda K., Hinz B. (2014). Cannabinoids increase lung cancer cell lysis by lymphokine-activated killer cells via upregulation of ICAM-1. Biochem. Pharmacol..

[203] Sharma M., Hudson J.B., Adomat H., Guns E., Cox M.E. (2014). In Vitro Anticancer Activity of Plant-Derived Cannabidiol on Prostate Cancer Cell Lines. Pharmacol. Pharm..

[204] Fogli S., Nieri P., Chicca A., Adinolfi B., Mariotti V., Iacopetti P., Breschi M.C., Pellegrini S. (2006). Cannabinoid derivatives induce cell death in pancreatic MIA PaCa-2 cells via a receptor-independent mechanism. FEBS Lett..

[205] Hinshaw D.C., Shevde L.A. (2019). The tumor microenvironment innately modulates cancer progression. Cancer Res..

[206] Truffi M., Sorrentino L., Corsi F. (2020). Fibroblasts in the Tumor Microenvironment. Neuropilin.

[207] Nagl L., Horvath L., Pircher A., Wolf D. (2020). Tumor Endothelial Cells (TECs) as Potential Immune Directors of the Tumor Microenvironment—New Findings and Future Perspectives. Front. Cell Dev. Biol..

[208] Bernard J.J., Wellberg E.A. (2021). The Tumor Promotional Role of Adipocytes in the Breast Cancer Microenvironment and Macroenvironment. Am. J. Pathol..

[209] Sun R., Kong X., Qiu X., Huang C., Wong P.-P. (2021). The Emerging Roles of Pericytes in Modulating Tumor Microenvironment. Front. Cell Dev. Biol..

[210] Bejarano L., Jordāo M.J., Joyce J.A. (2021). Therapeutic Targeting of the Tumor Microenvironment. Cancer Discov..

[211] Baghban R., Roshangar L., Jahanban-Esfahlan R., Seidi K., Ebrahimi-Kalan A., Jaymand M., Kolahian S., Javaheri T., Zare P. (2020). Tumor microenvironment complexity and therapeutic implications at a glance. Cell Commun. Signal..

[212] Anderson N.M., Simon M.C. (2020). The tumor microenvironment. Curr. Biol..

[213] Deville S.S., Cordes N. (2019). The Extracellular, Cellular, and Nuclear Stiffness, a Trinity in the Cancer Resistome—A Review. Front. Oncol..

[214] Winkler J., Abisoye-Ogunniyan A., Metcalf K.J., Werb Z. (2020). Concepts of extracellular matrix remodelling in tumour progression and metastasis. Nat. Commun..

[215] LaGory E.L., Giaccia E.L.L.A.J. (2016). The ever-expanding role of HIF in tumour and stromal biology. Nature.

[216] Voron T., Colussi O., Marcheteau E., Pernot S., Nizard M., Pointet A.-L., Latreche S., Bergaya S., Benhamouda N., Tanchot C. (2015). VEGF-A modulates expression of inhibitory checkpoints on CD8+ T cells in tumors. J. Exp. Med..

[217] Kim J.M., Chen D.S. (2016). Immune escape to PD-L1/PD-1 blockade: Seven steps to success (or failure). Ann. Oncol..

[218] Pasquier J., Ghiabi P., Chouchane L., Razzouk K., Rafii S., Rafii A. (2020). Angiocrine endothelium: From physiology to cancer. J. Transl. Med..

[219] Halama A., Guerrouahen B.S., Pasquier J., Satheesh N.J., Suhre K., Rafii A. (2017). Nesting of colon and ovarian cancer cells in the endothelial niche is associated with alterations in glycan and lipid metabolism. Sci. Rep..

[220] D’Arcangelo E., Wu N.C., Cadavid J.L., McGuigan A.P. (2020). The life cycle of cancer-associated fibroblasts within the tumour stroma and its importance in disease outcome. Br. J. Cancer.

[221] Ippolito L., Morandi A., Taddei M.L., Parri M., Comito G., Iscaro A., Raspollini M.R., Magherini F., Rapizzi E., Masquelier J. (2019). Cancer-associated fibroblasts promote prostate cancer malignancy via metabolic rewiring and mitochondrial transfer. Oncogene.

[222] Ippolito L., Morandi A., Giannoni E., Chiarugi P. (2019). Lactate: A Metabolic Driver in the Tumour Landscape. Trends Biochem. Sci..

[223] Giannoni E., Bianchini F., Masieri L., Serni S., Torre E., Calorini L., Chiarugi P. (2010). Reciprocal Activation of Prostate Cancer Cells and Cancer-Associated Fibroblasts Stimulates Epithelial-Mesenchymal Transition and Cancer Stemness. Cancer Res..

[224] Sahai E., Astsaturov I., Cukierman E., DeNardo D.G., Egeblad M., Evans R.M., Fearon D., Greten F.R., Hingorani S.R., Hunter T. (2020). A framework for advancing our understanding of cancer-associated fibroblasts. Nat. Rev. Cancer.

[225] Fiori M.E., Di Franco S., Villanova L., Bianca P., Stassi G., De Maria R. (2019). Cancer-associated fibroblasts as abettors of tumor progression at the crossroads of EMT and therapy resistance. Mol. Cancer.

[226] Linares J., Marín-Jiménez J.A., Badia-Ramentol J., Calon A. (2021). Determinants and Functions of CAFs Secretome during Cancer Progression and Therapy. Front. Cell Dev. Biol..

[227] Comito G., Giannoni E., Segura C.P., Barcellos-De-Souza P., Raspollini M.R., Baroni G., Lanciotti M., Serni S., Chiarugi P. (2013). Cancer-associated fibroblasts and M2-polarized macrophages synergize during prostate carcinoma progression. Oncogene.

[228] Comito G., Iscaro A., Bacci M., Morandi A., Ippolito L., Parri M., Montagnani I., Raspollini M.R., Serni S., Simeoni L. (2019). Lactate modulates CD4+ T-cell polarization and induces an immunosuppressive environment, which sustains prostate carcinoma progression via TLR8/miR21 axis. Oncogene.

[229] Harrell C.R., Markovic B.S., Fellabaum C., Arsenijevic A., Djonov V., Volarevic V. (2018). Molecular mechanisms underlying therapeutic potential of pericytes. J. Biomed. Sci..

[230] Bose A., Barik S., Banerjee S., Ghosh T., Mallick A., Majumdar S.B., Goswami K.K., Bhuniya A., Banerjee S., Baral R. (2013). Tumor-Derived Vascular Pericytes Anergize Th Cells. J. Immunol..

[231] Lengyel E., Makowski L., DiGiovanni J., Kolonin M.G. (2018). Cancer as a Matter of Fat: The Crosstalk between Adipose Tissue and Tumors. Trends Cancer.

[232] Fasshauer M., Blüher M. (2015). Adipokines in health and disease. Trends Pharmacol. Sci..

[233] Dirat B., Bochet L., Dabek M., Daviaud D., Dauvillier S., Majed B., Wang Y.Y., Meulle A., Salles B., Le Gonidec S. (2011). Cancer-Associated Adipocytes Exhibit an Activated Phenotype and Contribute to Breast Cancer Invasion. Cancer Res..

[234] Attané C., Muller C. (2020). Drilling for Oil: Tumor-Surrounding Adipocytes Fueling Cancer. Trends Cancer.

[235] Chiurchiù V., Battistini L., Maccarrone M. (2015). Endocannabinoid signalling in innate and adaptive immunity. Immunology.

[236] Braile M., Marcella S., Marone G., Galdiero M., Varricchi G., Loffredo S. (2021). The Interplay between the Immune and the Endocannabinoid Systems in Cancer. Cells.

[237] Matias I., Pochard P., Orlando P., Salzet M., Pestel J., Di Marzo V. (2002). Presence and regulation of the endocannabinoid system in human dendritic cells. JBIC J. Biol. Inorg. Chem..

[238] López A.J.S., Román-Vega L., Tojeiro E.R., Giuffrida A., García-Merino A. (2014). Regulation of cannabinoid receptor gene expression and endocannabinoid levels in lymphocyte subsets by interferon-β: A longitudinal study in multiple sclerosis patients. Clin. Exp. Immunol..

[239] Castaneda J.T., Harui A., Roth M.D. (2017). Regulation of Cell Surface CB2 Receptor during Human B Cell Activation and Differentiation. J. Neuroimmune Pharmacol..

[240] Sugamura K., Sugiyama S., Nozaki T., Matsuzawa Y., Izumiya Y., Miyata K., Nakayama M., Kaikita K., Obata T., Takeya M. (2009). Activated Endocannabinoid System in Coronary Artery Disease and Antiinflammatory Effects of Cannabinoid 1 Receptor Blockade on Macrophages. Circulation.

[241] Klein T.W. (2005). Cannabinoid-based drugs as anti-inflammatory therapeutics. Nat. Rev. Immunol..

[242] Jean-Gilles L., Braitch M., Latif M.L., Aram J., Fahey A.J., Edwards L.J., Robins R.A., Tanasescu R., Tighe P.J., Gran B. (2015). Effects of pro-inflammatory cytokines on cannabinoid CB1and CB2receptors in immune cells. Acta Physiol..

[243] Basu S., Dittel B.N. (2011). Unraveling the complexities of cannabinoid receptor 2 (CB2) immune regulation in health and disease. Immunol. Res..

[244] Ma Y., Ren Y., Dai Z.-J., Wu C.-J., Ji Y.-H., Xu J. (2017). IL-6, IL-8 and TNF-α levels correlate with disease stage in breast cancer patients. Adv. Clin. Exp. Med..

[245] Hirano T. (2020). IL-6 in inflammation, autoimmunity and cancer. Int. Immunol..

[246] Kienzl M., Kargl J., Schicho R. (2020). The Immune Endocannabinoid System of the Tumor Microenvironment. Int. J. Mol. Sci..

[247] Staiano R.I., Loffredo S., Borriello F., Iannotti F., Piscitelli F., Orlando P., Secondo A., Granata F., Lepore M.T., Fiorelli A. (2015). Human lung-resident macrophages express CB1 and CB2 receptors whose activation inhibits the release of angiogenic and lymphangiogenic factors. J. Leukoc. Biol..

[248] Glodde N., Jakobs M., Bald T., Tüting T., Gaffal E. (2015). Differential role of cannabinoids in the pathogenesis of skin cancer. Life Sci..

[249] Gruber T., Robatel S., Kremenovic M., Bäriswyl L., Gertsch J., Schenk M. (2021). Cannabinoid Receptor Type-2 in B Cells Is Associated with Tumor Immunity in Melanoma. Cancers.

[250] Ravi J., Elbaz M., Wani N.A., Nasser M.W., Ganju R.K. (2016). Cannabinoid receptor-2 agonist inhibits macrophage induced EMT in non-small cell lung cancer by downregulation of EGFR pathway. Mol. Carcinog..

[251] Gaffal E., Kemter A.M., Scheu S., Dantas R.L., Vogt J., Baune B., Tüting T., Zimmer A., Alferink J. (2020). Cannabinoid Receptor 2 Modulates Maturation of Dendritic Cells and Their Capacity to Induce Hapten-Induced Contact Hypersensitivity. Int. J. Mol. Sci..

[252] Qiu C., Yang L., Wang B., Cui L., Li C., Zhuo Y., Zhang L., Zhang S., Zhang Q., Wang X. (2019). The role of 2-arachidonoylglycerol in the regulation of the tumor-immune microenvironment in murine models of pancreatic cancer. Biomed. Pharmacother..

[253] Suk K.-T., Mederacke I., Gwak G.-Y., Cho S.W., Adeyemi A., Friedman R., Schwabe R.F. (2016). Opposite roles of cannabinoid receptors 1 and 2 in hepatocarcinogenesis. Gut.

[254] Xiang W., Shi R., Kang X., Zhang X., Chen P., Zhang L., Hou A., Wang R., Zhao Y., Zhao K. (2018). Monoacylglycerol lipase regulates cannabinoid receptor 2-dependent macrophage activation and cancer progression. Nat. Commun..

[255] Yin J., Kim S.S., Choi E., Oh Y.T., Lin W., Kim T.-H., Sa J.K., Hong J.H., Park S.H., Kwon H.J. (2020). ARS2/MAGL signaling in glioblastoma stem cells promotes self-renewal and M2-like polarization of tumor-associated macrophages. Nat. Commun..

[256] Ghosh S., Preet A., Groopman J., Ganju R. (2006). Cannabinoid receptor CB2 modulates the CXCL12/CXCR4-mediated chemotaxis of T lymphocytes. Mol. Immunol..

[257] Taylor L., Christou I., Kapellos T.S., Buchan A., Brodermann M.H., Gianella-Borradori M., Russell A., Iqbal A.J., Greaves D.R. (2015). Primary Macrophage Chemotaxis Induced by Cannabinoid Receptor 2 Agonists Occurs Independently of the CB2 Receptor. Sci. Rep..

[258] Montecucco F., Burger F., Mach F., Steffens S. (2008). CB2 cannabinoid receptor agonist JWH-015 modulates human monocyte migration through defined intracellular signaling pathways. Am. J. Physiol. Circ. Physiol..

[259] Raborn E.S., Marciano-Cabral F., Buckley N.E., Martin B.R., Cabral G.A. (2007). The Cannabinoid Delta-9-tetrahydrocannabinol Mediates Inhibition of Macrophage Chemotaxis to RANTES/CCL5: Linkage to the CB2 Receptor. J. Neuroimmune Pharmacol..

[260] Steffens S., Veillard N.R., Arnaud C., Pelli G., Burger F., Staub C., Zimmer A., Frossard J.-L. (2005). Fran Low dose oral cannabinoid therapy reduces progression of atherosclerosis in mice. Nature.

[261] Pietrovito L., Iozzo M., Bacci M., Giannoni E., Chiarugi P. (2020). Treatment with Cannabinoids as a Promising Approach for Impairing Fibroblast Activation and Prostate Cancer Progression. Int. J. Mol. Sci..

[262] Gęgotek A., Rybałtowska-Kawałko P., Skrzydlewska E. (2017). Rutin as a Mediator of Lipid Metabolism and Cellular Signaling Pathways Interactions in Fibroblasts Altered by UVA and UVB Radiation. Oxidative Med. Cell. Longev..

[263] Bobrov M.Y., Bezuglov V.V., Khaspekov L.G., Illarioshkin S.N., Novosadova E.V., Grivennikov I.A. (2017). Expression of Type I Cannabinoid Receptors at Different Stages of Neuronal Differentiation of Human Fibroblasts. Bull. Exp. Biol. Med..

[264] Solinas M., Massi P., Cantelmo A., Cattaneo M., Cammarota R., Bartolini D., Cinquina V., Valenti M., Vicentini L., Noonan D. (2012). Cannabidiol inhibits angiogenesis by multiple mechanisms. Br. J. Pharmacol..

[265] Picardi P., Ciaglia E., Proto M., Pisanti S. (2014). Anandamide inhibits breast tumor-induced angiogenesis. Transl. Med. UniSa.

[266] Pisanti S., Borselli C., Oliviero O., Laezza C., Gazzerro P., Bifulco M. (2006). Antiangiogenic activity of the endocannabinoid anandamide: Correlation to its tumor-suppressor efficacy. J. Cell. Physiol..

[267] Luo C.-K., Chou P.-H., Ng S.-K., Lin W.-Y., Wei T.-T. (2021). Cannabinoids orchestrate cross-talk between cancer cells and endothelial cells in colorectal cancer. Cancer Gene Ther..

[268] Zong Y., Zhou X., Cheng J., Yu J., Wu J., Jiang C. (2017). Cannabinoids Regulate the Diameter of Pericyte-Containing Retinal Capillaries in Rats. Cell. Physiol. Biochem..

[269] Bellocchio L., Cervino C., Vicennati V., Pasquali R., Pagotto U. (2008). Cannabinoid Type 1 Receptor: Another Arrow in the Adipocytes’ Bow. J. Neuroendocr..

[270] Gasperi V., Fezza F., Pasquariello N., Bari M., Oddi S., Agrò A.F., Maccarrone M. (2006). Endocannabinoids in adipocytes during differentiation and their role in glucose uptake. Experientia.

[271] Mazier W., Saucisse N., Gatta-Cherifi B., Cota D. (2015). The Endocannabinoid System: Pivotal Orchestrator of Obesity and Metabolic Disease. Trends Endocrinol. Metab..

[272] Wu Q., Li B., Li Z., Li J., Sun S., Sun S. (2019). Cancer-associated adipocytes: Key players in breast cancer progression. J. Hematol. Oncol..

[273] Wu Q., Sun S., Li Z., Yang Q., Li B., Zhu S., Wang L., Wu J., Yuan J., Yang C. (2018). Tumour-originated exosomal miR-155 triggers cancer-associated cachexia to promote tumour progression. Mol. Cancer.

[274] Guzmán M., Duarte M.J., Blázquez C., Ravina J., Rosa M.C., Galve-Roperh I., Sanchez C., Velasco G., González-Feria L. (2006). A pilot clinical study of Δ9-tetrahydrocannabinol in patients with recurrent glioblastoma multiforme. Br. J. Cancer.

[275] Singh Y., Bali C. (2013). Cannabis Extract Treatment for Terminal Acute Lymphoblastic Leukemia with a Philadelphia Chromosome Mutation. Case Rep. Oncol..

[276] Foroughi M., Hendson G., Sargent M.A., Steinbok P. (2011). Spontaneous regression of septum pellucidum/forniceal pilocytic astrocytomas—possible role of Cannabis inhalation. Child’s Nerv. Syst..

[277] Twelves C., Sabel M., Checketts D., Miller S., Tayo B., Jove M., Brazil L., Short S.C. (2021). A phase 1b randomised, placebo-controlled trial of nabiximols cannabinoid oromucosal spray with temozolomide in patients with recurrent glioblastoma. Br. J. Cancer.

[278] Torres S., Lorente M., Rodríguez-Fornés F., Hernández-Tiedra S., Salazar M., García-Taboada E., Barcia J., Guzmán M., Velasco G. (2011). A Combined Preclinical Therapy of Cannabinoids and Temozolomide against Glioma. Mol. Cancer Ther..

[279] Borrelli F., Pagano E., Romano B., Panzera S., Maiello F., Coppola D., De Petrocellis L., Buono L., Orlando P., Izzo A.A. (2014). Colon carcinogenesis is inhibited by the TRPM8 antagonist cannabigerol, a Cannabis-derived non-psychotropic cannabinoid. Carcinogenesis.

[280] Chen L., Chen H., Li Y., Li L., Qiu Y., Ren J. (2015). Endocannabinoid and ceramide levels are altered in patients with colorectal cancer. Oncol. Rep..

[281] Kargl J., Haybaeck J., Stančić A., Andersen L., Marsche G., Heinemann A., Schicho R. (2012). O-1602, an atypical cannabinoid, inhibits tumor growth in colitis-associated colon cancer through multiple mechanisms. J. Mol. Med..

[282] Hinz B., Ramer R. (2018). Anti-tumour actions of cannabinoids. Br. J. Pharmacol..

[283] Blázquez C., González-Feria L., Álvarez L., Haro A., Casanova M.L., Guzmán M. (2004). Cannabinoids Inhibit the Vascular Endothelial Growth Factor Pathway in Gliomas. Cancer Res..

[284] Kogan N.M., Blázquez C., Álvarez L., Gallily R., Schlesinger M., Guzmán M., Mechoulam R. (2006). A Cannabinoid Quinone Inhibits Angiogenesis by Targeting Vascular Endothelial Cells. Mol. Pharmacol..

[285] Honarmand M., Namazi F., Mohammadi A., Nazifi S. (2018). Can cannabidiol inhibit angiogenesis in colon cancer?. Comp. Haematol. Int..

[286] Ladin D.A., Soliman E., Griffin L., Van Dross R. (2016). Preclinical and Clinical Assessment of Cannabinoids as Anti-Cancer Agents. Front. Pharmacol..

[287] Kisková T., Mungenast F., Suváková M., Jäger W., Thalhammer T. (2019). Future Aspects for Cannabinoids in Breast Cancer Therapy. Int. J. Mol. Sci..

[288] Verykiou S., Alexander M., Edwards N., Plummer R., Chaudhry B., Lovat P., Hill D.S. (2018). Harnessing autophagy to overcome mitogen-activated protein kinase kinase inhibitor-induced resistance in metastatic melanoma. Br. J. Dermatol..

[289] Massi P., Vaccani A., Ceruti S., Colombo A., Abbracchio M.P., Parolaro D. (2003). Antitumor Effects of Cannabidiol, a Nonpsychoactive Cannabinoid, on Human Glioma Cell Lines. J. Pharmacol. Exp. Ther..

[290] Aguado T., Carracedo A., Julien B., Velasco G., Milman G., Mechoulam R., Alvarez L., Guzmán M., Galve-Roperh I. (2007). Cannabinoids Induce Glioma Stem-like Cell Differentiation and Inhibit Gliomagenesis. J. Biol. Chem..

[291] Singer E., Judkins J., Salomonis N., Matlaf L., Soteropoulos P., McAllister S.D., Soroceanu L. (2015). Reactive oxygen species-mediated therapeutic response and resistance in glioblastoma. Cell Death Dis..

[292] Kogan N.M., Schlesinger M., Peters M., Marincheva G., Beeri R., Mechoulam R. (2007). A Cannabinoid Anticancer Quinone, HU-331, Is More Potent and Less Cardiotoxic than Doxorubicin: A Comparative in Vivo Study. J. Pharmacol. Exp. Ther..

[293] Caffarel M.M., Andradas C., Perez-Gomez E., Guzmán M., Sanchez C. (2012). Cannabinoids: A new hope for breast cancer therapy?. Cancer Treat. Rev..

